# The Role of Interventional Radiology in the Diagnosis and Treatment of Gynecological Conditions

**DOI:** 10.3390/tomography12090131

**Published:** 2026-09-11

**Authors:** Arkadiusz Kacała, Julia Rydzek, Marta Zawadzka, Wiktoria Andryszkiewicz, Maria Wojtaszek, Marta Żywica, Anna Myroshnychenko, Daniel Soliński, Krzysztof Dyś, Tomasz Fuchs, Maciej Guziński

**Affiliations:** 1 Department of Radiology, Wroclaw Medical University, 50-367 Wroclaw, Poland; 2 Department of General, Interventional and Neuroradiology, Wroclaw University Hospital, 50-367 Wroclaw, Poland; 3 Faculty of Medicine, Wroclaw Medical University, 50-367 Wroclaw, Poland; 4 Clinical Department of Obstetrics and Gynecology, University Center of Obstetrics and Gynecology, Wroclaw Medical University, 50-367 Wroclaw, Poland

**Keywords:** interventional radiology, uterine artery embolization, placenta accreta spectrum, splenic artery aneurysm, pregnancy, cesarean scar pregnancy, gynecologic malignancy, tumor embolization

## Abstract

Interventional radiology (IR) has reshaped contemporary management strategies for selected gynecological and obstetric disorders by introducing minimally invasive, organ-sparing therapeutic alternatives to conventional surgical approaches. This review examines the current applications of IR in the treatment of symptomatic uterine fibroids, pelvic congestion syndrome, placenta accreta spectrum, cesarean scar pregnancy, and selected gynecological malignancies requiring palliative or preoperative embolization. In addition, selected vascular conditions in pregnancy, including visceral and cerebral aneurysms, are discussed due to their high-risk clinical profile and growing relevance for endovascular management. These entities were chosen because of their substantial clinical relevance, risk of severe complications, and the expanding body of evidence supporting endovascular management. Techniques such as uterine artery embolization and ovarian vein embolization are associated with high technical success and meaningful symptom reduction, while simultaneously decreasing hospitalization time, perioperative morbidity, and convalescence compared with traditional surgery. In complex obstetric scenarios—including placenta accreta spectrum and splenic artery aneurysm—prophylactic balloon occlusion and endovascular embolization may significantly limit life-threatening hemorrhage and improve maternal prognosis in appropriately selected patients when implemented within a multidisciplinary setting; for splenic artery aneurysm in particular, this evidence currently derives from case reports and small case series rather than comparative trials. Imaging modalities, including ultrasonography, computed tomography, magnetic resonance imaging, and angiography, are integral to diagnosis, patient qualification, procedural planning, and post-intervention monitoring. Although existing data generally confirm the safety and efficacy of interventional techniques, important limitations persist, including heterogeneous study populations, relatively small cohorts, and insufficient long-term reproductive follow-up. Greater standardization of clinical indications and procedural protocols, ongoing technological advancement, and well-designed multicenter trials are required to consolidate the evidence base. In gynecologic oncology, embolization techniques may provide effective hemorrhage control and facilitate surgical treatment in selected patients. In addition, uterine artery embolization has emerged as an important uterus-preserving adjunct in the management of cesarean scar pregnancy, although long-term reproductive (fertility) outcome data remain limited. IR is becoming an increasingly important component of modern gynecological care, supporting individualized, uterus-preserving, and less invasive therapeutic strategies; fertility preservation specifically is an important goal that, for several of the indications discussed, remains incompletely proven by long-term reproductive-outcome data.

## Simple Summary

Interventional radiology uses small tubes and instruments guided by imaging, rather than open surgery, to treat certain gynecological and pregnancy-related conditions. This review summarizes how these image-guided techniques are used for uterine fibroids, pelvic vein disorders, dangerous placental attachment, cesarean scar pregnancy, some cancers of the female reproductive organs, and rare but serious artery bulges during pregnancy. Compared with conventional surgery, these procedures can shorten hospital stays and recovery, reduce blood loss, and often preserve the uterus; for several conditions, evidence also suggests a benefit for future fertility, although long-term reproductive data remain limited. The review highlights current evidence, benefits, limitations, and areas needing further research to help guide safer, more personalized care for women.

## 1. Introduction

Over the last three decades, interventional radiology (IR) has evolved from a primarily diagnostic subspecialty into a source of catheter-based, image-guided therapeutic alternatives to open surgery across gynecological and obstetric care [1]. Conventional surgical management—hysterectomy, myomectomy, and open vascular reconstruction—remains effective for many benign and life-threatening gynecological conditions [2], but carries considerable tissue trauma, blood loss, and, for some procedures, irreversible loss of the uterus or fertility, driving demand for the less invasive, organ-preserving alternatives IR is positioned to provide [2,3,4]. The effectiveness of IR is closely linked to advances in diagnostic imaging. Accurate patient qualification and meticulous procedural planning depend on high-resolution imaging modalities, including ultrasonography (USG), computed tomography (CT), magnetic resonance imaging (MRI), and angiography [5,6]. Ultrasound remains the first-line imaging technique in gynecological diagnostics owing to its accessibility, safety, and cost-efficiency. It plays a pivotal role in identifying uterine fibroids, evaluating pelvic venous insufficiency, and detecting placental abnormalities. Doppler assessment of vascular flow is particularly valuable in conditions such as PCS [7,8]. MRI provides superior soft-tissue contrast and precise anatomical characterization. In fibroid assessment, MRI enables detailed evaluation of lesion number, size, vascularization, and spatial relationships, thereby facilitating appropriate qualification for embolization. In PAS, MRI complements ultrasound by enabling assessment of placental invasion depth and adjacent organ involvement [9,10]. CT, although used cautiously in women of reproductive age due to radiation exposure, remains important in emergency situations and in the evaluation of vascular pathology such as splenic artery aneurysm. Angiography serves both diagnostic and therapeutic purposes, allowing direct visualization of vascular anatomy and immediate intervention. Together, these modalities constitute the cornerstone of safe and effective interventional management [6,11]. In addition to the above-mentioned gynecological and obstetric conditions, increasing attention has been directed toward vascular pathologies occurring during pregnancy, in which interventional radiology may play a life-saving role [12]. Among these, visceral artery aneurysms (VAAs)-particularly splenic artery aneurysm (SAA)-represent rare but clinically significant entities due to their strong association with pregnancy and high risk of rupture [13]. Cerebral aneurysms, although less directly influenced by pregnancy, constitute an important cause of hemorrhagic stroke and require careful multidisciplinary management [14]. Although cerebral aneurysms are not gynecologic in origin, their inclusion is warranted here because rupture during pregnancy constitutes a maternal neurovascular emergency that demands the same multidisciplinary, radiation-conscious, endovascular-first approach emphasized throughout this review, notwithstanding substantial overlap with neurointerventional practice. These conditions further expand the scope of IR in modern obstetric care [15]. Beyond benign gynecological diseases and obstetric emergencies, interventional radiology is increasingly applied in gynecologic oncology and abnormal implantation disorders [16].

Palliative and preoperative embolization may effectively control tumor-related hemorrhage and facilitate surgery, while uterine artery embolization has become an important adjunct in the management of cesarean scar pregnancy. These emerging applications further broaden the role of minimally invasive image-guided therapies in contemporary gynecological care. Although IR’s role in individual gynecologic and obstetric conditions has previously been reviewed separately, no single narrative synthesis has yet integrated the elective, benign indications addressed by IR (uterine fibroids, pelvic congestion syndrome) with the acute obstetric and oncologic applications of IR (placenta accreta spectrum, cesarean scar pregnancy, hemorrhage from gynecologic malignancy, and visceral or cerebral aneurysms in pregnancy) within one evidence-graded, multidisciplinary framework; this review addresses that gap [17].

## 2. Methods

### 2.1. Search Strategy and Study Selection

This narrative review employed a structured literature search and screening process informed by the Preferred Reporting Items for Systematic Reviews and Meta-Analyses (PRISMA 2020) framework, in order to ensure transparency and reproducibility of study identification. Given the broad clinical scope of the conditions addressed, the marked heterogeneity of the included study designs, and the absence of prospective protocol registration, the identified evidence is synthesized and presented as a narrative review rather than as a formal systematic review or meta-analysis. The review aimed to identify and synthesize, through a structured but non-systematic search and selection process, the available evidence regarding the diagnostic and therapeutic applications of interventional radiology in gynecological and obstetric conditions. This structured approach was adopted to enhance transparency and reproducibility, while the narrative-review designation was retained given the heterogeneity of the included conditions and the absence of prospective protocol registration. The literature search was performed using PubMed, EMBASE, Cochrane Library, and Web of Science databases for articles published between January 2000 and April 2026. The search strategy combined Medical Subject Headings (MeSHs) and free-text keywords related to interventional radiology in gynecology and obstetrics, including “interventional radiology”, “uterine artery embolization”, “uterine fibroids”, “leiomyoma”, “pelvic congestion syndrome”, “pelvic venous disorders”, “ovarian vein embolization”, “placenta accreta spectrum”, “prophylactic balloon occlusion”, “visceral artery aneurysm”, “splenic artery aneurysm”, “cerebral aneurysm”, “pregnancy”, “endovascular management”, “cesarean scar pregnancy”, “scar ectopic pregnancy”, “gynecologic malignancy”, “cervical cancer”, “pelvic tumor embolization”, “palliative embolization”, and “preoperative embolization”. A representative full PubMed search string, illustrating how these terms were combined for one of the six clinical topics, was (“uterine artery embolization” [MeSH] OR “uterine artery embolization” [tiab] OR “UAE” [tiab]) AND (“leiomyoma” [MeSH] OR “uterine fibroids” [tiab] OR “leiomyoma” [tiab]) AND (“humans” [MeSH] AND English[lang]) AND (“2000/01/01” [PDAT]:“2026/04/30”[PDAT]). Analogous condition-specific strings, built from the same MeSHs and free-text terms listed above and combined with the same human/English-language/date filters, were run for each of the remaining five clinical topics; database-specific syntax (Emtree headings and the/exp and/de qualifiers in EMBASE, the Cochrane Library’s Medical Subject Headings search field, and Web of Science’s Topic (TS=) field) was substituted for the PubMed MeSH/tiab syntax shown above while keeping the same underlying concepts, filters, and date range.

Studies were eligible if they met the following criteria: (1) original clinical studies involving human participants; (2) randomized controlled trials, prospective or retrospective cohort studies, case–control studies, or large case series; (3) studies evaluating diagnostic or therapeutic applications of interventional radiology in gynecological or obstetric conditions; and (4) full-text articles published in English. Review articles, editorials, conference abstracts, letters to the editor, animal studies, duplicate publications, and studies without relevant clinical outcome data were excluded.

The primary database search yielded a total of 2436 records across the six clinical topics:

- 684 records for uterine artery embolization;
- 342 records for pelvic congestion syndrome;
- 451 records for placenta accreta spectrum;
- 196 records for cesarean scar pregnancy;
- 331 records for gynecological malignancies requiring embolization;
- 432 records for vascular aneurysms in pregnancy.

Following automated and manual removal of duplicates, 1782 unique records remained for title and abstract screening. Subsequently, 486 full-text articles were assessed for eligibility. Of these, 338 studies were excluded because of inappropriate study design, insufficient outcome reporting, duplicate patient populations, or lack of clinical relevance to the scope of this review.

**Figure 1:**
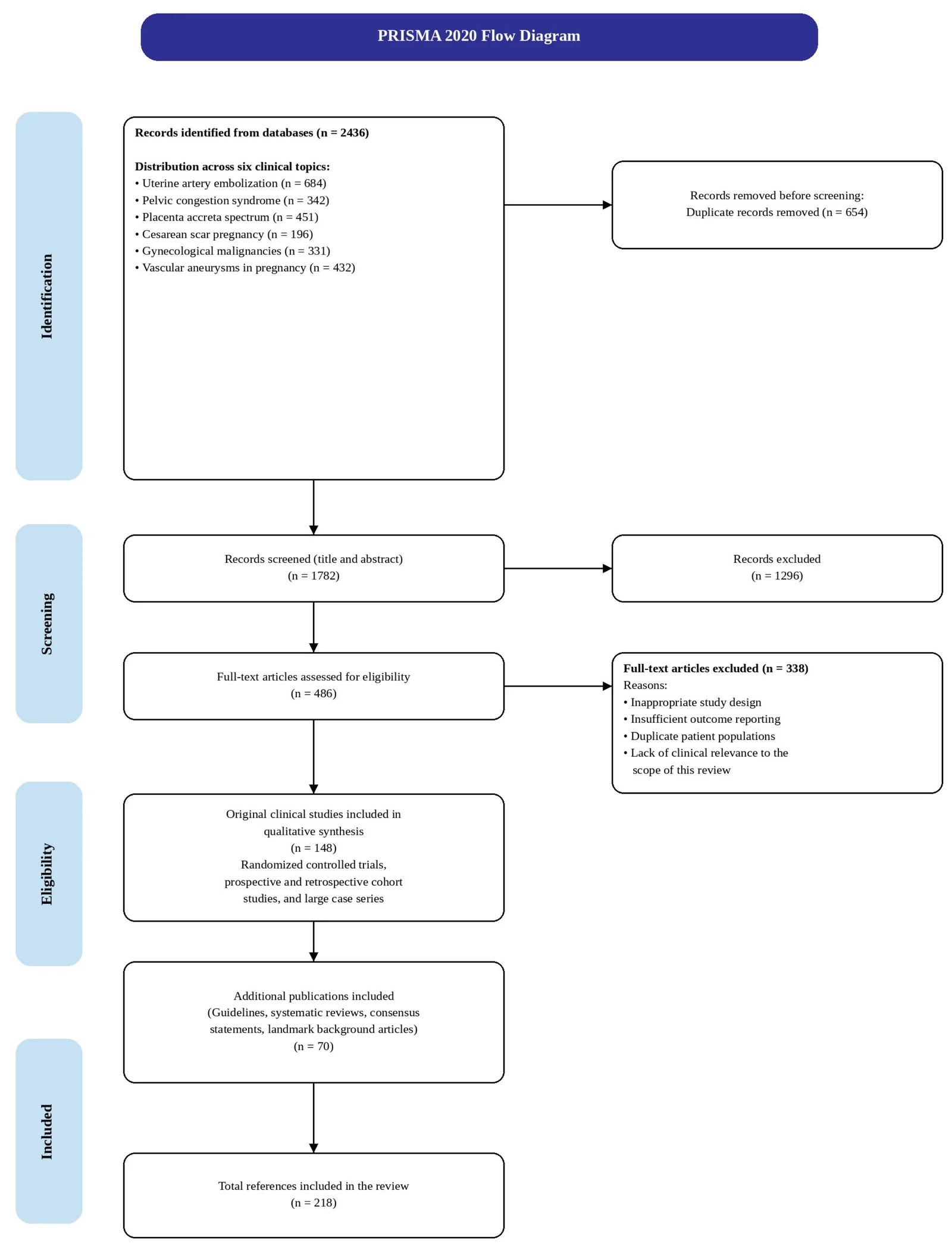
PRISMA 2020 flow diagram of the literature search and study selection process. A total of 2436 records were identified across six clinical topics in PubMed, EMBASE, Cochrane Library, and Web of Science (January 2000–April 2026). After removal of 654 duplicates, 1782 records were screened by title and abstract, and 486 full-text articles were assessed for eligibility. Ultimately, 148 original clinical studies were included in the qualitative synthesis. An additional 70 publications (guidelines, systematic reviews, consensus statements, and landmark background articles), identified through targeted non-systematic searches and used solely for narrative context, are shown separately, giving a final bibliography of 218 references.

Ultimately, 148 original clinical studies, including randomized controlled trials, prospective and retrospective cohort studies, and large case series, were included for detailed data extraction. In addition, 70 publications comprising international guidelines, systematic reviews, consensus statements, and landmark background articles were incorporated to provide comprehensive clinical context, resulting in a final bibliography of 218 references. These 70 contextual sources were identified through targeted, non-systematic searches rather than the structured screening process described above; they were used exclusively for narrative framing, guideline context, and background discussion, were not part of the systematically screened evidence base informing the clinical synthesis, and are accordingly shown separately in the PRISMA flow diagram (Figure 1), outside the structured screening pathway, although all are included in the reference list.

The study selection process is summarized in the PRISMA flow diagram (Figure 1).

### 2.2. Data Extraction and Quality Assessment

Data extraction was performed independently by the co-authors using a standardized digital form, with final verification by the senior supervising authors.

Titles, abstracts, and full-text articles were screened independently by two reviewers. Any disagreements regarding study eligibility or extracted data were resolved through discussion until consensus was reached. The following information was systematically extracted from each eligible study: study characteristics (design, publication year, country), patient demographics (age, parity, gestational age where applicable), clinical indications (fibroid burden, chronic pelvic pain characteristics, placental invasion depth, aneurysm dimensions and locations), intervention specifications (embolic materials, catheter types, balloon placement locations, radiation-reduction protocols), clinical/technical outcome measures, follow-up duration, and reproductive/fertility data.

The methodological quality of the included randomized controlled trials was evaluated using the Cochrane Risk of Bias tool. For observational cohort and case–control studies, the Newcastle–Ottawa Scale was employed, assessing selection parameters, comparability of cohorts, and the ascertainment of clinical outcomes or exposure. The quality of included systematic reviews and meta-analyses was assessed using the AMSTAR-2 (A MeaSurement Tool to Assess systematic Reviews) instrument.

While individual primary studies were assessed using these dedicated tools, a pooled summary of risk of bias was not compiled due to the high heterogeneity of study designs across the six clinical entities reviewed. Most historical randomized trials comparing interventional techniques to open surgery demonstrated a moderate risk of performance bias due to the inherent impossibility of blinding operators and patients to surgical versus percutaneous techniques. Observational data showed variable risk depending on the standardization of follow-up intervals and reporting of long-term reproductive outcomes. The certainty of evidence was not formally rated using the GRADE framework; study design hierarchy, cohort size, and the consistency of outcomes across centers were nonetheless used qualitatively by the authors to gauge the overall reliability of the evidence base underlying each narrative statement, rather than to derive a formal quantitative weighting. In summary, across the six clinical entities, the randomized trials identified (concentrated in the uterine fibroid literature comparing UAE with surgery) were generally at moderate risk of performance and detection bias, an inherent consequence of the impossibility of blinding patients or operators to a percutaneous versus an open surgical procedure, but were mostly at low risk of selection and attrition bias. Observational cohort and case–control studies, assessed with the Newcastle–Ottawa Scale, were more variable: single-center retrospective series describing balloon occlusion in PAS and embolization for splenic artery aneurysm or acute tumor hemorrhage most often scored in the moderate-to-high risk-of-bias range, chiefly because of unclear comparability of cohorts (absence of a concurrent surgical comparison group) and inconsistent, non-standardized ascertainment of long-term reproductive and functional outcomes; larger multicenter registries and prospective cohorts, concentrated in the fibroid and PCS literature, scored more favorably on these same domains. The systematic reviews and meta-analyses appraised with AMSTAR-2 varied chiefly in whether a protocol was registered in advance and whether publication bias was formally assessed, with more recent (2022–2025) reviews generally meeting more AMSTAR-2 critical domains than older ones. A study-by-study risk-of-bias listing was not compiled into a supplementary table in this revision; the qualitative, domain-level summary above reflects the pattern observed across the appraised literature.

### 2.3. Data Synthesis and Analysis

Given the broad clinical scope of this review and the distinct pathophysiological natures of the conditions evaluated (benign gynecological conditions versus high-risk obstetric vascular emergencies), a qualitative narrative synthesis approach was mandated rather than a formal quantitative meta-analysis. Studies were segmented and analyzed based on their primary clinical target:

- Uterine artery embolization for symptomatic fibroids;
- Ovarian and internal iliac vein embolization for pelvic congestion syndrome;
- Prophylactic endovascular balloon occlusion for placenta accreta spectrum;
- Interventional management of cesarean scar pregnancy;
- Palliative and preoperative embolization in gynecological malignancies;
- Endovascular treatment (coiling, stenting or embolization) of visceral and cerebral aneurysms during pregnancy.

Data derived from high-quality clinical trials, multicenter registries, and societal consensus statements were prioritized to formulate the definitive comparison tables (Table 1, Table 2, Table 3, Table 4 and Table 5) and the conceptual workflow presented in the manuscript. Where homogeneous quantitative outcomes were available across multiple studies (such as technical success percentages or reintervention rates), ranges were extracted and presented; no formal weighted-mean or other pooled quantitative estimate was calculated, consistent with the narrative (non-meta-analytic) design of this review. Reporting bias (such as publication bias via funnel plots) was not formally assessed statistically due to the descriptive review design; however, potential bias—including the overreporting of successful endovascular management in rare emergency scenarios—was qualitatively factored into the final discussion and synthesis of clinical limitations.

The review protocol was not prospectively registered; as a narrative rather than systematic review, it was not eligible for registration in systematic-review registries such as PROSPERO. This is nonetheless acknowledged as a limitation with respect to the a priori transparency of the review process. Because of the marked clinical and methodological heterogeneity of the included studies, a formal GRADE assessment and quantitative meta-analysis were not performed.

## 3. The Role of Interventional Radiology in the Treatment of Uterine Fibroids

### 3.1. Epidemiology and Clinical Significance of Uterine Fibroids

Uterine fibroids, referred to as leiomyomas or myomas, are frequently occurring tumors found in the uterus. These fibroids consist of both smooth muscle and fibroblast elements, along with a significant amount of fibrous extracellular matrix, all of which play a role in their development. The characteristics of fibroids vary widely regarding their biological behavior, size, position, and the symptoms they produce. Leiomyomas are benign smooth-muscle tumors and do not undergo malignant transformation; they should be distinguished from the rare, pathologically distinct entities of smooth-muscle tumor of uncertain malignant potential (STUMP) and leiomyosarcoma, which are separate diagnoses defined by specific histopathological criteria rather than a progression from conventional leiomyoma. The likelihood of developing fibroids is influenced by ethnicity; African American women face a greater risk of encountering fibroids at an earlier age compared to white women, as well as experiencing more severe manifestations of the condition [18]. Twenty-five percent of women in their reproductive years show signs of uterine fibroids, with about a quarter of these women needing hospital care because of the condition’s seriousness. The prevalence of this illness is often underestimated since numerous women do not show symptoms, and these symptoms develop slowly; as a result, many cases go undiagnosed. The precise prevalence of uterine fibroids can differ based on the population studied and the diagnostic criteria used; however, the occurrence of uterine fibroids in reproductive-aged women varies between 5.4 percent and 77 percent [19]. Even though the majority of women dealing with fibroids do not show symptoms, around 30% will experience significant issues such as unusual bleeding from the uterus, anemia, tiredness, chronic vaginal discharge, and pain during periods [20]. Other symptoms include protrusion of the abdomen, pain during intercourse, dysfunctions of bladder/bowel leading to urinary incontinence/retention, pain, and constipation. It is also associated with reproductive issues like impaired fertility, conceiving complications, and adverse obstetric outcomes [21]. Both infertility and recurrent early pregnancy loss are symptoms associated with fibroids. Uterine fibroids that do not change the structure of the fallopian tubes are not likely to affect fertility, at least not by altering the transport of sperm, eggs, or embryos through the tubes. Fibroids are also linked to negative pregnancy results, including premature delivery, abnormal fetal positioning, placental abruption, and difficult labor. The likelihood of these complications increases for individuals with several large fibroids (greater than 4–5 cm) that are located behind the placenta [22].

### 3.2. Imaging Diagnostics for Fibroids

USG remains first-line for the diagnosis of leiomyomas due to its high sensitivity and specificity. It is also associated with its availability, ease of use, absence of radiation exposure, and relatively low cost [23]. It can be conducted either through the vagina (transvaginal scan (TVS)) or through the abdomen (transabdominal scan (TAS)). Typically, transvaginal ultrasound is considered more effective than transabdominal ultrasound for the majority of pelvic issues [24]. TVS is particularly more effective at identifying small leiomyomas and is advantageous in cases involving retroverted or retroflexed uteri. Additionally, TVS is beneficial for patients who have a lot of gas in the bowel, those who cannot adequately fill their bladder, and individuals who are obese, making TAS difficult to perform. The main drawback of TVS is that it has a limited scanning depth, which means larger or pedunculated myomas may not be visible with high-frequency probes that have short focal lengths [25,26].

In ultrasound assessments, leiomyomas typically show up as distinct, solid, round, hypoechoic lumps that create varying degrees of acoustic shadowing. Nonetheless, the degree of calcification or the presence of fibrous tissue can lead to leiomyomas displaying different levels of echogenicity, typically being hyperechogenic or isoechogenic. Calcifications appear as bright spots with shadowing effects. Occasionally, leiomyomas might exhibit anechoic areas due to advancing necrosis. In challenging situations where leiomyomas are small and similar in echogenicity to the surrounding myometrium, the only detectable sign on ultrasound may be a protrusion in the shape of the uterus [27].

Nevertheless, the precision of ultrasound imaging is constrained by factors such as substantial body size, a significantly enlarged uterus, and the presence of shadowing caused by multiple fibroids. Additionally, ultrasound is not a dependable method for evaluating the blood flow to fibroids, which is essential for forecasting the effectiveness of UAE [20]. However, MRI can provide information on the number of fibroids, their size, vascularization, relationship with the endometrial cavity and serosal surface, and boundaries with normal myometrium; therefore, it is currently seen as the most effective imaging method for examining myomas [28]. Even though it comes at a much higher cost, MRI remains the most precise tool for identifying fibroids, boasting a sensitivity rate of 99%. Furthermore, MRI assists in evaluating the structure of the uterus and ovaries, and it is useful in preparing for myomectomy procedures. On T1 and T2 MRI scans, leiomyomas show up as regions of low or moderate signal with distinct borders [29,30].

MRI offers excellent contrast for soft tissues and a comprehensive field of view. This imaging technique is particularly useful for mapping fibroids in advance of surgery or minimally invasive procedures, as well as for differentiating between International Federation of Gynecology and Obstetrics (FIGO) classifications, such as distinguishing Type 3 from Type 4, or deep Type 2 from Types 2 through 5. Additionally, it delivers vital insights concerning blood flow, signs of tissue degeneration, and characteristics that help differentiate fibroids from adenomyosis or adenomyomas [26,31,32,33].

#### Patient Selection for Uterine Artery Embolization

A proper assessment of patients is essential for achieving optimal clinical outcomes and reducing the risk of complications following UAE. Suitable candidates should be females experiencing symptoms from uterine fibroids, with no other pelvic conditions, and who do not intend to become pregnant later on [34]. Choosing patients for UAE involves evaluating their symptoms, medical background, the quantity, size, and position of the leiomyomas or other uterine issues, the patient’s desire for future pregnancies, and their treatment choices. Although transvaginal ultrasound is the preferred initial imaging method, MRI is useful to examine the severity of the condition, rule out different diseases, and consider the pros and cons of UAE compared to other treatment methods. Certain conditions absolutely rule out the use of UAE, such as being pregnant, having an active infection, or potential cancers of the uterus, cervix, or surrounding areas [35,36].

### 3.3. Uterine Artery Embolization

UAE was first launched in 1995 as a less invasive treatment choice that preserves the uterus for premenopausal women dealing with symptomatic fibroids. The method is carried out through a transfemoral percutaneous technique to reach both internal iliac arteries. Once the catheter’s position in the internal iliac artery is verified, a guide wire is inserted into the uterine artery, followed by the catheter being positioned over the guide wire [37]. When inserting a catheter into the uterine artery, it is important to avoid rough handling and unnecessary use of a guidewire, as these can lead to vascular damage and spasms at the point where the uterine artery begins, ultimately decreasing the chances of success and the effectiveness of the treatment. The uterine artery can be accessed using a main catheter; however, it is best to insert the microcatheter while minimizing movement or use of the guidewire to reduce the risk of spasms. In these situations, employing a high-flow microcatheter with a wider lumen is advisable to facilitate the effective delivery of the embolic material, since the size of the spherical embolization particles utilized for UAE is typically 500 μm or larger. Subsequently, angiography is conducted to evaluate the blood supply and dimensions of the fibroid prior to the injection of the embolic material [38].

UAE involves either a short-term or long-term blockage of the uterine arteries by using materials that are safe for the body to cut off the blood flow to fibroids. It entails temporarily blocking the arteries that supply blood to the uterus by means of biocompatible materials [39].

A variety of embolic substances have been utilized in UAE, each with distinct characteristics such as polyvinyl alcohol (PVA) particles, gelatin foam, n-butyl-2 cyanoacrylate, and coils, among other options. It is important to highlight that PVA is commonly employed, but it is regarded as a permanent solution along with n-butyl-2 cyanoacrylate, while gelatin foam is viewed as a temporary option for embolization [40]. A catheter is used to deliver these particles into the blood vessels of the uterus while using fluoroscopy for guidance. The success of embolization is indicated when the contrast material administered with fluoroscopic support remains still in the upward segment of the uterine artery for roughly five heartbeats. Since blood flow can resume after the embolization procedure, it is necessary to carry out angiography about five minutes post-injection to confirm that the uterine fibroids no longer show signs of staining and that the upper uterine artery is intact [37].

**Figure 2:**
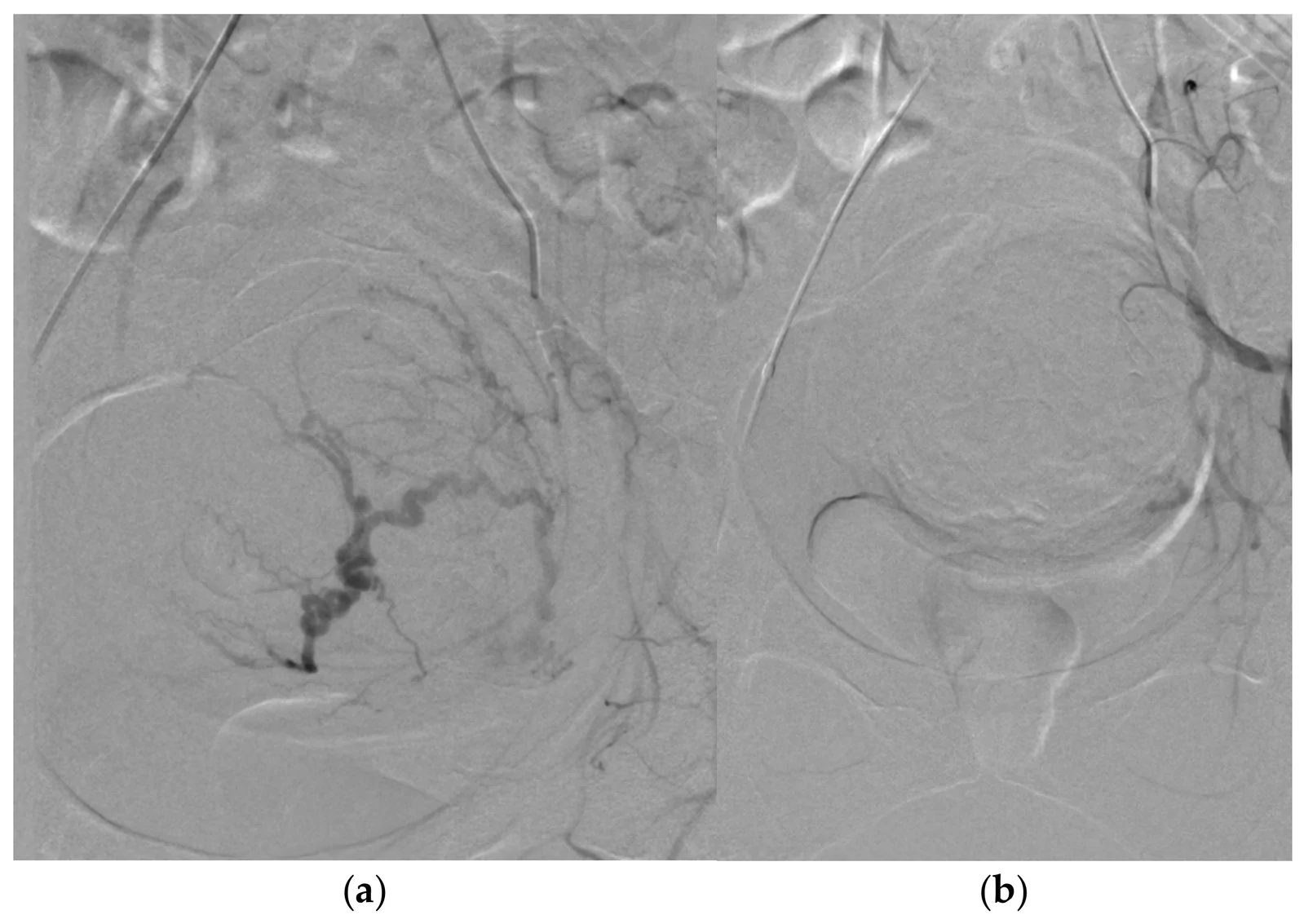
Endovascular embolization of a symptomatic uterine fibroid. (**a**) Pre-procedural digital subtraction angiography (DSA) of the left uterine artery demonstrates a markedly enlarged, tortuous main trunk feeding a large (8.5 cm), hypervascularized, round uterine fibroid with intense tumor stain and abnormal neoangiogenesis. (**b**) Post-procedural angiography following bilateral uterine artery embolization (UAE) reveals complete occlusion of the tumor-feeding branches and stasis of contrast medium within the main uterine artery trunk, with preservation of normal myometrial perfusion.

The blockage of blood vessels reduces the flow of blood and nutrient delivery to fibroid areas, triggering a series of cellular reactions that result in cell death and changes in tissue structure. The death of cells effectively diminishes fibroids and results in a reduction in the uterine size, granting patients some relief from their symptoms (Figure 2).

UAE is safe and effective, and has the advantages of shorter hospital stay and recovery time as compared to surgery [41]. However, the method also has certain drawbacks that hinder its broader use, like post-embolization syndrome, which may involve complete cessation of menstruation, minor impairment of ovarian function (notably in women over 45), and the potential need for further intervention or a subsequent hysterectomy. Other than postembolization syndrome, which is generally regarded as a normal part of healing, the complications that are most commonly noted include short-term or long-term amenorrhea and ongoing vaginal discharge. Complications that are noted less often include fibroid expulsion (FE), prolonged or persistent pain, infections, urinary retention, and injuries related to access [42]. Other uncommon issues consist of fatalities from blood clots in the lungs or infections, accidental blockage caused by leiomyosarcoma, tissue death in the uterus, buttocks, or labia, and abnormal connections between the bladder and uterus [43]. UAE has specific contraindications, such as ongoing pregnancy, active infections, and suspected cancers of the uterus, cervix, or adjacent structures. On the other hand, UAE offers several benefits, including reduced blood loss and shorter surgical durations [44].

Even though UAE is quite successful in alleviating symptoms, like minimizing bleeding and the size of fibroids, the possibility of needing another procedure exists: 15 to 20 percent after a successful embolization and as much as 50 percent when the infarction is not fully achieved [28].

Myomectomy is a method of treatment for individuals with uterine fibroids that preserves the uterus. This procedure can be done through an abdominal approach, using laparoscopy with or without the aid of robotics or through a hysteroscopic method [45]. While myomectomy and UAE both aim to preserve the uterus for women with fibroids who wish to retain the option of future pregnancy, the success rates of pregnancies following myomectomy are better than those after UAE, showing increased instances of clinical pregnancy and live births, along with reduced occurrences of spontaneous abortion, abnormal placentation, preterm labor, and malpresentation [3]. UAE is linked to a notable benefit over myomectomy regarding initial complications, the need for readmission, length of hospital stays, and costs associated with hospitalization. Nevertheless, UAE could have certain drawbacks in comparison to myomectomy when considering long-term results like the need for further interventions, hysterectomy, and changes in the severity of the condition [2]. The rate of repeat procedures for UAE is greater than that for myomectomy [44].

Hysterectomy has long been the main method for treating problematic fibroids and is still the only conclusive treatment option available. The procedure offers a complete solution for women experiencing troublesome fibroids who do not plan to maintain their ability to have children, leading to full alleviation of symptoms and enhanced quality of life [27,46]. In contrast to hysterectomy, UAE offers a shorter hospital stay and allows patients to resume regular activities sooner, but it also comes with a greater chance of requiring further procedures. Considering the hormonal functions of the uterus, any surgical alterations to this organ might lead to variations in the sex hormone levels produced by the ovaries, due to changes in their blood supply. Consequently, the choice of surgical technique significantly influences patient outcomes, and current evidence does not yet provide a definitive answer on the most effective way to manage uterine fibroids [47]. In contrast to hysterectomy and myomectomy, UAE leads to a notably shorter hospital stay, a quicker return to everyday activities, and a lower chance of needing a blood transfusion.

### 3.4. Limitations and Research Gaps

Research conducted over a longer period has indicated that UAE provides lasting symptom relief, frequently removing the necessity for more invasive surgical procedures like hysterectomy [48]. The likelihood of needing further interventions after an average of five to nine years is low, roughly between 15 and 25 percent. Various studies have highlighted a notable enhancement in different aspects of HRQoL (health-related quality of life) [48,49].

Pregnancy and live birth are possible for patients after UAE. However, the extent to which UAE affects fertility remains unknown and needs further study [41]. Possible consequences for the reproductive organs following UAE may involve ovarian dysfunction linked to reduced blood supply, damage to the fallopian tubes from infections leading to infertility and instances of uterine rupture during delivery. There have also been observations of higher miscarriage rates and placental issues, such as abnormal placental attachments; therefore, UAE could potentially influence various outcomes after pregnancy has been confirmed [37]. Even though UAE is recognized as a successful and less invasive solution for treating uterine fibroids, there are worries regarding its effect on a woman’s ovarian reserve for those who wish to conceive in the future. It has been suggested that accidental blocking of the utero-ovarian collateral blood vessels during the UAE procedure may disrupt the blood flow to the ovaries, potentially resulting in a reduction in ovarian reserve [38]. Tulandi and colleagues indicated that UAE negatively affects ovarian reserve. Nevertheless, more recent research utilizing anti-Müllerian hormone (AMH) to assess ovarian reserve has shown no significant decrease in ovarian reserve following UAE. More recent large-scale systematic reviews, meta-analyses, and prospective cohorts published between 2023 and 2025 have since extended this evidence base with larger, more contemporary reproductive-outcome data than the earlier studies discussed above [50,51,52]. Nonetheless, because many individual underlying studies remain relatively limited in size, additional data is needed to reach a definitive conclusion [53,54,55].

There is a need for more randomized studies focusing on evaluating the desire for pregnancy and including women who wish to conceive in the future. There are constraints in the available research regarding the pregnancy rates and outcomes for women of reproductive age who wish to become pregnant after having UAE for various reasons. Many individuals in the current studies and case reports possess different factors that complicate straightforward analysis or comparison. These factors include older maternal age, issues with the uterine wall, past miscarriages, previous surgeries on the uterus, different techniques used by interventional radiologists, uncertain intentions to conceive and carry a pregnancy to term, and other unidentified infertility issues [56]. Future research could concentrate on clearly defined (sub)groups that might gain more advantage from a non-surgical intervention, such as women with a significant fibroid load, those with anemia, or individuals who have undergone previous myomectomy, as these groups are thought to have an increased risk of complications during surgery and could particularly benefit from UAE. The same is applicable for large (giant) fibroids, which can be successfully managed through UAE; however, the limited existing evidence suggests a relatively higher chance of complications and the need for further procedures [35].

**Table 1:** Comparison of treatment options for uterine fibroids: myomectomy, hysterectomy and uterine artery embolization.

Feature	Uterine Artery Embolization	Myomectomy	Hysterectomy
Mechanism of Action	Closing the blood supply to the fibroids	Removal of only the fibroids	Removal of the entire uterus
Type of procedure	Minimally invasive, percutaneous	Surgical (abdominal, laparoscopic, hysteroscopic)	Surgical removal of the uterus
Fertility preservation	Yes (but limited data; possible impairment)	Yes (best option for future pregnancy)	No
Effectiveness	Effective symptom relief (bleeding, fibroid size)	Effective, especially for symptomatic fibroids	Definitive treatment (complete symptom resolution)
Hospital stay and recovery	Shorter hospital stay, faster recovery	Longer than UAE	Longest recovery
Need for further procedures	Relatively frequent	Less frequent	Not needed
Best candidates	Women seeking less invasive option, not prioritizing fertility	Women desiring future pregnancy	Women not wishing to preserve fertility
References	[3,41]	[3,45]	[46,47]

## 4. Pelvic Congestion Syndrome

### 4.1. Pathophysiology and Clinical Presentation

Pelvic pain that lasts more than six months is considered chronic pelvic pain (CPP). It is a frequent but often misdiagnosed condition that affects over 40% of women. In up to half cases, no definite reason can be determined even after extensive testing, such as laparoscopy. PCS, also known as pelvic venous insufficiency (PVI), might be responsible for as many as 30% of CPP cases, but it is often overlooked. The dull, persistent pain that is exacerbated by standing, sexual activity and before menstruation is caused by reduced blood flow in the pelvic veins. It primarily targets premenopausal women, particularly after childbirth [57].

Other vague symptoms may also occur, such as swelling of the vulva, abnormal vaginal discharge, a frequent urge to urinate, rectal discomfort, pain in the back or hips, varicosities affecting the vulva, perineum, or legs, persistent genital arousal, bloating, nausea, headaches, fatigue, and low mood [57].

The effects extend beyond just physical symptoms. In contrast to short-term pain, PCS is persistent, frequently resistant to treatment, and has the potential to cause problems like depression, central sensitization, and long-term impairment. It is frequently associated with mental health issues like past trauma, PTSD, and anxiety. Due to the frequent misdiagnosis caused by overlapping symptoms with gynecological, gastrointestinal, urinary, musculoskeletal, and psychosomatic disorders, many patients spend years looking for a diagnosis [57]. The location or severity of discomfort might not always correspond to conditions such as endometriosis or adhesion. The way the nervous system handles signals from internal organs may be the reason why regular sensations feel painful. As a result, a lot of women with CPP do not receive a definitive diagnosis. Without a thorough history, non-structural causes like irritable bowel syndrome may be missed [58].

Among the possible reasons of CPP one of the most challenging are Pelvic Venous Disorders (PeVDs). Symptoms arise from three main mechanisms: ovarian vein reflux, left iliac vein compression (May–Thurner syndrome), and left renal vein compression (Nutcracker syndrome). Blood in the ovarian vein usually flows upward, but reflux happens when valves fail, frequently as a result of vein dilatation caused by pregnancy. The left iliac vein is compressed in May–Thurner syndrome, which raises pelvic pressure and causes reflux and varicose veins. The left renal vein is squeezed in Nutcracker syndrome, which causes blood to flow backwards into the ovarian vein, causing it to dilate [59]. To standardize classification, the SVP (Systems–Varices–Pathophysiology) system was introduced [60].

### 4.2. Imaging Diagnostics

The best method for identifying pelvic venous illnesses is venography. It allows direct assessment of venous flow, pressure measurements, and provocation tests [59]. Enlarged, twisted veins with an irregular diameter and contrast pooling are signs of severe congestion. This approach is invasive, takes time, and exposes women to radiation, even if it is successful [61]. Findings like an ovarian vein diameter greater than 6 mm, prolonged contrast retention (more than 20 s), venous stasis in the pelvis or lower extremities, and reflux are used to diagnose PVD. In addition to enabling prompt treatment, it also gives a thorough overview of venous anatomy prior to embolization and is able to identify reflux more effectively than certain noninvasive techniques. It has a sensitivity of about 91% and a specificity of roughly 89% [62]. However, needless exposure to ionizing radiation should be taken into consideration because a large number of CPP patients are premenopausal women. USG, CT, and MRI are common alternatives in assessing CPP and suspected PVI [63].

Magnetic resonance venography is extremely effective at identifying pelvic, ovarian, and hypogastric vein insufficiency in women with PCS. Hypogastric veins are particularly vulnerable (up to 100%), although the low specificity causes a high number of false positive findings. MRI provides more comprehensive data and helps differentiate PCS from other disorders such as endometriosis, adenomyosis, uterine fibroids, or gynecologic malignancy [64].

The initial screening test for assessing pelvic veins is an ultrasound. Doppler measures blood flow and permits dynamic testing using the Valsalva maneuver; whereas, B-mode examines anatomy and rules out tumors. Transperineal, transabdominal, or transvaginal procedures are all possible. TVUS (transvaginal ultrasonography) is preferable for gynecological evaluation, while TAUS (transabdominal ultrasonography) and transperineal techniques provide a more comprehensive view of the vasculature [65].

Pelvic venography verified the results in 187 cases (95.9%) of the 195 patients who had at least one TVUS sign of PeVD. Dilated arcuate veins in the myometrium communicating with pelvic varicosities were confirmed in 96.4% of patients. When the right standards are applied, TVUS offers a great level of diagnostic accuracy, making it a crucial first-line tool [66]. On TVUS, PVD can manifest as reflux, sluggish flow (<3 cm/s), dilated or tortuous ovarian veins, or expanded pelvic veins. Significant diagnostic indicators are reflux lasting longer than one second, venous dilatation during the Valsalva technique, and unusual flow patterns such as reversal or cross-pelvic shunting [67].

In most cases, CT is used less frequently than USG or MRI for diagnosing PVD because of the radiation exposure and higher cost, particularly in premenopausal women. Among the diagnostic criteria are an ovarian vein greater than 8 mm and at least four ipsilateral pelvic veins, one of which must be larger than 4 mm, with no interfering masses. CT can evaluate areas that are more difficult to reach with ultrasound, such as the left renal or common iliac vein, as well as reflux [68].

### 4.3. Interventional Treatment

**Figure 3:**
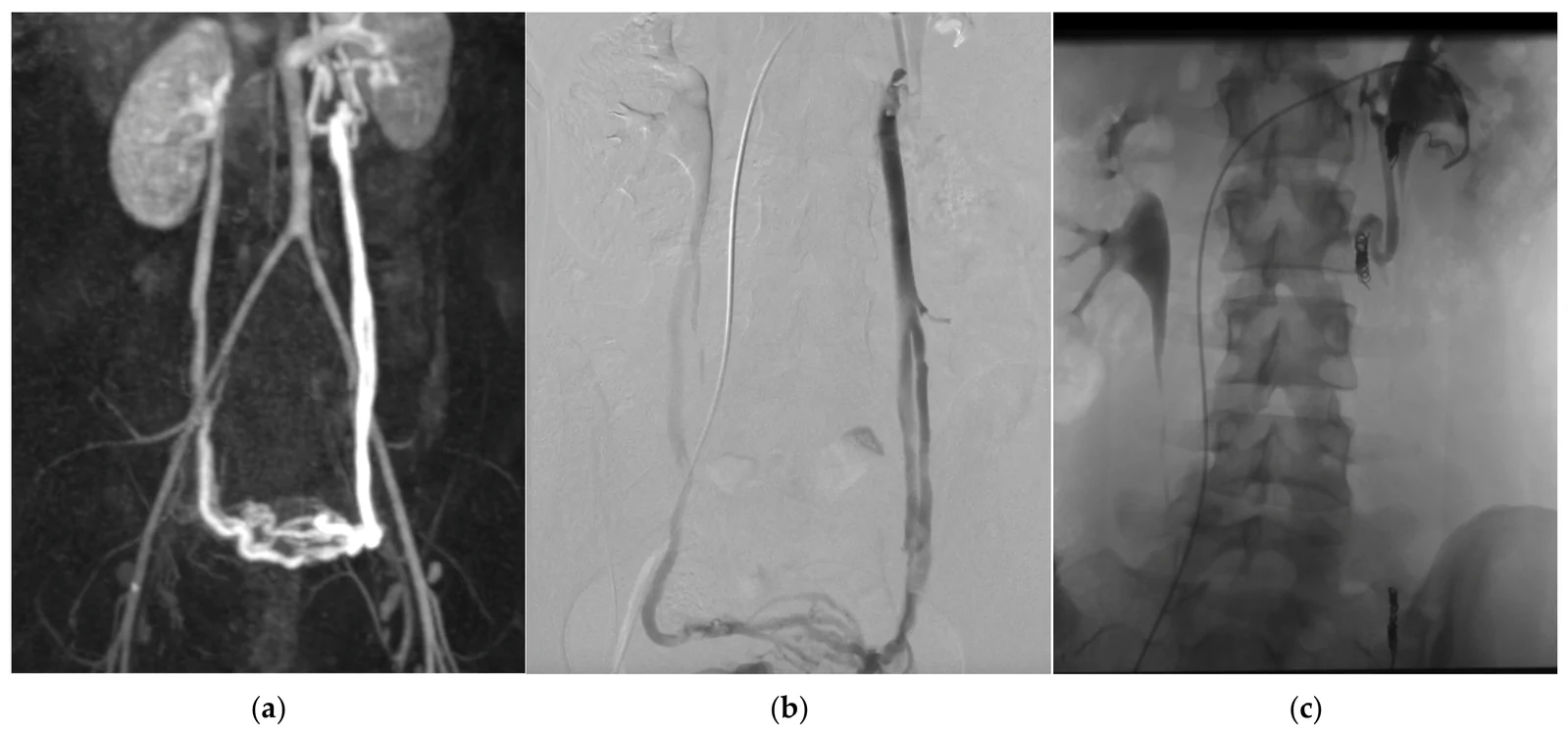
Imaging and endovascular treatment of left ovarian vein insufficiency with left-to-right parametrial reflux. (**a**) Dynamic contrast-enhanced MR (TWIST sequence) demonstrates dilatation of the left ovarian vein with pathological reflux and cross-pelvic venous drainage to the right side within the parametrial plexus. (**b**) Selective venography after catheter placement in the left renal vein confirms left ovarian vein insufficiency with retrograde flow and extensive pelvic varicosities, including left-to-right shunting. (**c**) Post-embolization image following coil occlusion of the left ovarian vein shows successful exclusion of the refluxing vessel with resolution of pathological cross-pelvic flow.

Several trials have demonstrated that ovarian vein embolization has a high rate of technical and clinical success. Based on limited to moderate data, coil or plug embolization and/or sclerotherapy are recommended. With a wide range of strategies (coils, plugs, liquid agents, or combinations), technical effectiveness is consistently excellent (96–100%) with a noticeable decrease in pain and associated symptoms. The most notable, but rare, complication is coil migration. Some evidence suggest a potential improvement in fertility outcomes following treatment, with low recurrence and reintervention rates [69]. Particularly in individuals with more severe illness, treatment of both the ovarian and internal iliac veins may result in better outcomes. The procedure is often carried out through the femoral or jugular veins. A catheter is inserted into the ovarian vein, reflux is verified with contrast, and embolic substances are used to stop aberrant blood flow [70]. Although embolization is a successful therapy for PCS, untreated or newly incompetent pelvic veins can cause a recurrence of symptoms. One of the cases demonstrates that reflux can originate from other veins, such as the uncommon median sacral vein, even after a successful ovarian vein embolization. To locate these alternative routes, repeat venography is essential. Targeted embolization of the affected vein can once again result in symptom relief [71] (Figure 3).

### 4.4. Controversies and Challenges

Dilated pelvic veins and ovarian vein reflux are also observed in asymptomatic, particularly multiparous, women, and chronic pelvic pain is multifactorial; imaging evidence of venous insufficiency should therefore be interpreted as one contributory factor rather than proof of a direct causal relationship, with PCS diagnosed only after correlating imaging with the clinical picture and excluding other causes of chronic pelvic pain. Management of PCS is complex and still debated, but it generally includes three main approaches: medical treatment, endovascular procedures, and surgery [72]. There is still a lot of discussion regarding the diagnosis, treatment, and long-term effects of PCS, especially on reproductive health. Pelvic blood flow and ovarian perfusion may be impacted by endovenous embolization, which may have an impact on ovarian reserve and menstrual cycle patterns [73]. In one of the studies, hormone levels (follicle-stimulating hormone (FSH), luteinizing hormone (LH), estradiol (E2), prolactin) were unaffected by embolization, but AMH showed a minor decline, particularly in younger individuals. While this could indicate a potential effect on the ovarian reserve, the majority of outcomes are within the anticipated physiological range, as AMH naturally declines with age [73].

**Table 2:** Comparison of imaging modalities for the diagnosis of PCS: Doppler ultrasound, CT, MRI, and phlebography.

Modality	Role	Criteria	Advantages	Limitations
USG Doppler (TVUS/TAUS/transperineal)	First-line screening	- Dilated/tortuous ovarian or pelvic veins - Reflux > 1 s - Flow reversal or cross-pelvic shunting - Valsalva-induced dilation	- Non-invasive - No radiation - Dynamic (Valsalva) - Widely available - High accuracy when criteria applied	- Operator-dependent - Limited visualization of deep veins - May miss complex anatomy
CT	Adjunct/problem-solving	- Ovarian vein > 8 mm - ≥4 ipsilateral pelvic veins (≥1 > 4 mm) - Can show reflux, obstruction (e.g., renal/iliac veins)	- Good overview of anatomy - Evaluates areas inaccessible to US (renal, iliac veins)	- Ionizing radiation - Less preferred in premenopausal women - Higher cost vs. US
MRI	Comprehensive non-invasive evaluation	- Pelvic/ovarian/hypogastric vein insufficiency - High detection of hypogastric vein disease	- No radiation - Excellent soft tissue detail - Differentiates other causes (e.g., endometriosis, fibroids, malignancy)	- Lower specificity, false positives - More expensive, less available
Venography (Phlebography)	Gold standard + therapeutic planning	- Ovarian vein > 6 mm - Contrast retention > 20 s - Venous stasis - Reflux - Dilated, tortuous veins with pooling	- Direct visualization of flow - Pressure measurement - Provocation testing - Best for detecting reflux - Enables simultaneous treatment (embolization)	- Invasive - Time-consuming - Radiation exposure
References	[61,65]	[61,62]	[62,66]	[63,68]

## 5. Interventional Radiology in the Prevention of Placenta Accreta Hemorrhage

### 5.1. Placenta Accreta Spectrum—Clinical Significance and Prenatal Diagnosis

PAS is a pathological condition that happens during pregnancy where abnormal trophoblast invasion occurs leading to problems with placenta separation after delivery. The incidence of PAS is still rising globally as a result of increasing cesarean delivery rates [74]. PAS is one of the most life-threatening conditions during pregnancy, as the placenta cannot be detached from the uterine wall without inducing significant, massive hemorrhage which leads to high maternal morbidity and mortality [75,76]. Hemorrhage can cause hemodynamic instability and therefore patients frequently need a transfer to intensive care units and a blood transfusion. PAS can also lead to lower urinary tract trauma and sepsis, disseminated intravascular coagulation (DIC), and ultimately, death if it is not effectively managed [77]. The surgical management is required and in most cases hysterectomy is the typical treatment with potential complications such as hemorrhage, and injury to other pelvic organs. This form of treatment results in loss of future fertility; however, uterine preservation is not proposed often due to mentioned danger of fatal outcome. The prolonged complications include reoperation, persistent emotional and social problems and a lower quality of life [76,78].

The proper diagnosis and management improves the outcome. It is provided by a multidisciplinary team and includes planned delivery and proper surgery method [79]. Prenatal detection and risk stratification starts with an ultrasound—the first-line imaging technique for PAS, as it shows placental location, morphology, and abnormal blood flow. The ultrasound imaging technique include greyscale imaging, color Doppler imaging and three-dimensional power Doppler. This diagnostic method enables personalized surgical planning, which reduces the risk of complications from early delivery or invasive procedures, improving outcomes for both mother and baby [80,81]. The first ultrasound markers of PAS can be observed in the first trimester. The ultrasound finding most strongly associated with PAS is persistent placenta previa at the time of delivery, especially in patients who underwent cesarean section. Additional characteristic sonographic signs include placental lacunae, a loss of “clear zone”—a physiological hypoechoic zone between the placenta and the myometrium, thinning of the underlying myometrium, increased blood flow within the uterovesical or retroplacental area, invasion of placental tissue into the uterus and/or bladder, and the presence of bridging vessels. What is more, prominent color Doppler flow in the retroplacental space that can be observed together with abnormal bridging vessels has also been linked to PAS [76,82]. Since PAS usually happens in a previous cesarean scar, transvaginal ultrasound is important for the early recognition, monitoring and managing of this condition. PAS can be subdivided to placenta increta where chorionic villi penetrate deeply into the myometrium but do not reach the serosa and placenta percreta where the villi penetrate through the full thickness of the uterine wall, often reaching other organs localized in the pelvis such as the bladder. Unfortunately, no ultrasound sign is specific to identify the right diagnosis in these subtypes [80,83]. MRI is a useful diagnostic technique especially when ultrasound results are unclear or this method is limited. In the case of placenta increta or placenta percreta, MRI can be a supporting tool as it enables obstetricians to observe how deep the invasion actually is. Another advantage of MRI is that it shows a clear picture of how the placenta and surrounding pelvic structures are positioned. Although ultrasound is still the first-line imaging technique, MRI can be used for more complex cases and it contributes to more effective planning before the operation. The five main MRI signs that can be observed include uterine bulging, heterogeneous signal intensity, dark intraplacental bands on T2-weighted MRI, focal interruption of myometrium and tenting of bladder. All of the signs have presented high predicted accuracy for recognizing PAS [84,85,86].

### 5.2. Application of Intra-Vascular Balloons for Hemorrhage Prevention in Placenta Accreta Spectrum

It is crucial in the management of PAS to identify the safety and effectiveness of IR before recommending the surgical technique. The proper diagnosis is crucial in terms of preparing delivery, safe removal of the placenta reducing to minimum the risk of hemorrhage [80]. As mentioned before, the best surgical option for patients is cesarean section and following hysterectomy to control life-threatening blood loss. To perform this procedure safely, other surgical methods including interventional endovascular techniques are essential in these patients to control bleeding. These techniques are balloon occlusion and selective arterial embolization such as proximal ligation or embolization of the internal iliac or uterine arteries. Balloon occlusion can be described as placing a balloon catheter in a vessel during the preparation of the surgery and then inflating it after the delivery to limit blood loss and perform the procedure more safely. When a cesarean section is performed, balloon catheters are usually found on both sides in the common or internal iliac arteries [87,88]. Balloon occlusion of the internal iliac artery enables to decrease the blood supply of the uterine artery and offers more time during surgical management of PAS. It also lowers transfusion volumes demanded during the surgery [89]. The decision on whether to perform this procedure or not is made by a multidisciplinary team. The major criteria for the selection are how deeply the placenta invades, previous cesarean sections and a history of placenta previa. It is also important to include patient preference. There are several factors that determine how long the balloon catheter is used. The most important are how severe the case is, the anatomy of the patient and experience of a specialist who performs this procedure. After the cesarean section the balloon is removed if no severe blood loss is observed [90]. The occlusion of the abdominal aorta can be an alternative because occlusion of the internal iliac or uterine arteries may be less effective due to alternative blood vessels (collateral circulation) in the pelvis. In that case, even if the internal iliac or uterine arteries are blocked with balloons, blood can quickly flow through these other vessels and reach the uterus. This is the reason why blocking only the internal iliac arteries may not stop all bleeding. The balloons located in the abdominal aorta are more effective, since they are placed below the right renal artery and offer the complete blockage of pelvic blood supply [87,88,91].

The balloon occlusion procedure reduces the blood loss during the cesarean section; however, it involves side effects which should be recognized. The complication that can be observed may include ischemic necrosis of the lower limbs, thrombosis in the artery, bleeding at the puncture site, vessel injury, tissue and organ damage and acute renal failure. The thrombosis is probably caused by changes in the blood flow and higher risk to develop blood clots during pregnancy [90]. When the balloon is placed in the abdominal aorta, the complications include thrombosis and ischemia, uterine and bladder wall necrosis and neurological damage including femoral nerve injury [91]. Table 3 sums up the endovascular techniques used in the management of PAS.

Taking the side effects into consideration as well as previous studies, current evidence does not provide clear recommendations on whether or not to perform the procedure of prophylactic balloon occlusion in PAS management. Study results which describe its benefits remain mixed and inconsistent and the future research may be helpful to improve the patients stratification and decrease the level of potential complications during this procedure [90,92,93].

**Table 3:** The endovascular techniques used in the management of PAS.

Endovascular Technique	Placement	Mechanism of Action	Clinical Benefits	Possible Complications	References
Internal iliac artery balloon occlusion	Both internal iliac arteries	Reduces blood flow to uterine arteries during surgery	Reduces perioperative blood loss and transfusion needs	Thrombosis, ischemia of lower limbs, vascular injury	[89,90,92]
Abdominal aorta balloon occlusion	Abdominal aorta below renal arteries	Blocks blood supply to pelvis more completely	More effective pelvic blood flow control	Uterine/bladder wall necrosis, neural damage, thrombosis	[87,88,91]
Uterine artery embolization	Bilateral uterine arteries	Permanently blocks arterial blood flow to placenta	Controls postpartum hemorrhage; may preserve uterus	Postembolization syndrome, ischemia, hematoma	[94,95]

## 6. Interventional Radiology in Vascular Aneurysms During Pregnancy

### 6.1. Visceral Artery Aneurysms in Pregnancy

#### 6.1.1. Epidemiology and Clinical Relevance

**Figure 4:**
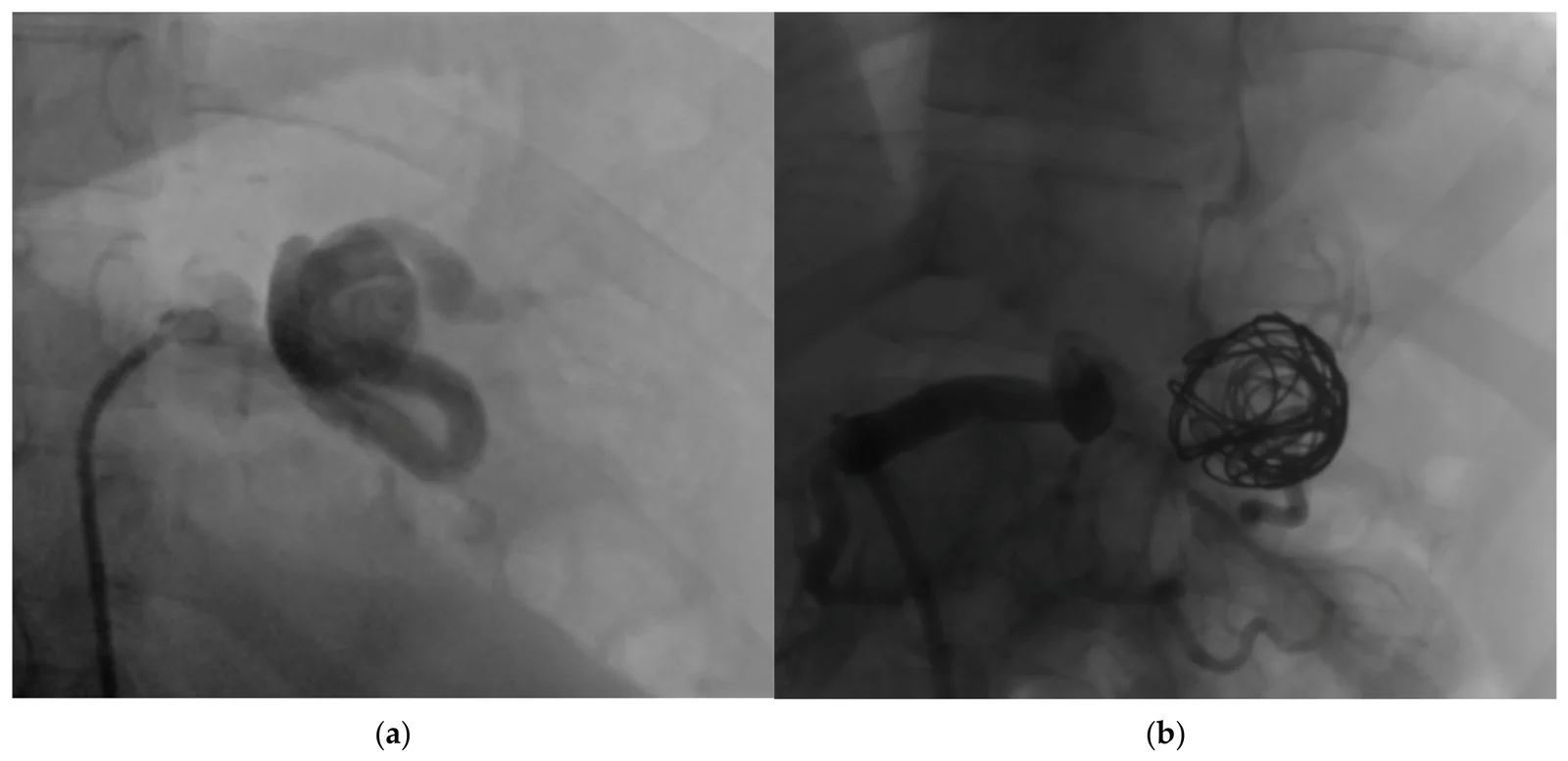
Endovascular management of a splenic artery aneurysm in a pregnant patient. (**a**) Pre-procedural angiography demonstrates a large (27 mm), wide-neck, high-flow aneurysm of the distal splenic artery with branches supplying the spleen arising from the aneurysm sac. (**b**) The aneurysm was secured prior to cesarean section using limited coil embolization, with only a small number of coils deployed to stabilize the aneurysm walls.

VAAs are rare occurring in about 0.01–0.1% of the population and can be described as a condition with a localized dilation of the splenic artery where the vessel diameter is increased by more than 50% compared to normal. The splenic artery is the most common site of VAAs, representing approximately 60–70% of cases. Other locations are hepatic artery, superior mesenteric artery and celiac artery. Even though visceral artery aneurysms are uncommon, they are highly life-threatening with mortality rates of up to 76% following rupture. The risk of rupture is associated with aneurysm size, rate of expansion and underlying disease [96,97,98]. SAA is the most clinically relevant due to its strong association with pregnancy and high rupture risk. Splenic artery aneurysms are associated with high mortality rates, estimated at 75% for mothers and up to 95% for fetuses [99,100] (Figure 4).

#### 6.1.2. Pathophysiology and Pregnancy-Related Risk Factors

The pathogenesis of VAAs in pregnancy has been studied. The development of the aneurysm is primarily caused by structural weakness of the arterial wall combined with increased hemodynamic pressure. During pregnancy, increased blood volume and cardiac output raise blood flow, thereby increasing wall stress and promoting vessel wall degeneration. The hormonal changes during pregnancy also play a crucial role in aneurysm development. Increased estrogen and progesterone may weaken the arterial wall, while higher relaxin levels may increase vessel elasticity resulting in splenic artery aneurysm dilation and rupture risk [101]. Pregnant women are also more prone to developing portal hypertension. In that case, hormones like aldosterone and rennin have also been linked to cause arterial wall thinning [102]. The splenic artery is particularly prone to aneurysm formation due to its tortuous anatomy, high blood flow, and relatively fragile arterial wall. In pregnancy, hormonal and haemodynamic changes further weaken the vessel wall, increasing the risk of aneurysm formation and rupture [98,103]. SAA most commonly occurs in the third trimester of pregnancy, as blood volume and cardiac output steadily increase during pregnancy reaching their highest levels in late pregnancy. Therefore, if surgery is needed, it should be performed in the first or second trimester [104,105].

#### 6.1.3. Risk of Rupture and Maternal–Fetal Outcomes

The sudden tearing of a visceral artery aneurysm is a critical and possibly fatal issue that can occur during pregnancy, posing risks to both the mother and the baby. Pregnancy is linked to a higher likelihood of rupture, likely due to alterations in blood flow and volume that occur during this time, changes in abdominal pressure from the growing uterus, and hormonal fluctuations that might weaken blood vessel walls [106].

The chance of death for the mother is between 70 and 75%; whereas, the risk of fatality for the unborn baby is significantly higher, at 90–95%. The final trimester and the initial period after childbirth pose significant risks. During this time, 24% to 45% of VAAs are known to burst; nonetheless, there have also been documented cases of rupture occurring in the first trimester of pregnancy [96].

Splenic artery aneurysms, also referred to as SAA, are the predominant type of visceral aneurysm and can pose a serious threat of rupture and dangerous complications when they exceed 3 cm, occur during pregnancy, or appear similar to pseudoaneurysms [107,108]. Rupture of SAA is the most dangerous among these aneurysms and can cause fatal hemorrhage and death [109].

#### 6.1.4. Imaging and Diagnosis

VAAs are usually asymptomatic and found incidentally during examinations performed for other indications [110,111]. As well SAA are often found by accident or during emergencies. VAAs are now being identified more often, which is linked to the regular application of MRI, CT, and ultrasound [112]. Ultrasound serves as the initial diagnostic test for individuals who may have a visceral aneurysm. This imaging technique also allows for a detailed examination of the structure of the aneurysm’s wall and sac, as well as an analysis of blood flow dynamics in that area [110,113]. CT is the leading technique used for imaging blood vessels at present. It assists in identifying the position, form, and dimensions of an aneurysm, evaluating the wall’s condition, and understanding its path and association with surrounding vessels. This test is frequently recommended for individuals who have experienced serious abdominal injuries or have undergone recent invasive procedures in the biliary tree that could result in aneurysms. Conditions such as kidney failure, lack of intravenous access, and sensitivity to a contrast agent prevent the use of computed tomography, making a diagnosis through this method unfeasible [110,114]. MRI offers extra benefits over CT: the contrast materials do not harm the kidneys and there is no exposure to ionizing radiation. Nevertheless, the presence of clips, stents, and coils can create artifacts that may complicate the evaluation or make it unfeasible [115].

#### 6.1.5. Interventional Management

Management of VAAs can be carried out through either an open surgical method or an endovascular technique. Open surgical interventions comprise options like ligation, complete or partial removal, patching (with vein or prosthetic material), primary repair, plication, and ex vivo repair [116,117]. Open surgical repair of vascular access anomalies is a reliable and long-lasting conventional treatment method. A key advantage of open surgery is the ability to visually assess the state of the affected organ. This means the necessity for revascularization can be verified, and outcomes can be evaluated during the procedure. However, due to improvements in and broader applications of endovascular therapy, open surgery is often chosen only when endovascular techniques are too challenging to carry out [116,117]. On the other hand, endovascular methods such as embolization, coiling, and the use of covered stents have gained popularity for treating VAAs due to their minimally invasive characteristics, expected quicker recovery periods, and typically reduced chances of complications and mortality [118]. The presence of complex bends in VAAs often limits options, making embolization the only practical choice [117]. The treatment of splenic artery aneurysms is influenced by their dimensions, position, and the symptoms they cause. Research indicates that transcatheter embolization is preferred due to its minimal risks for complications and mortality. Embolization is the standard treatment for stable patients; the procedure is exactly the same as discussed earlier [109]. For cases with multiple injuries, embolization of the proximal splenic artery is conducted between the dorsal and great pancreatic arteries, while distal embolization is intended for specific vascular injuries. There is no notable difference in the success of saving the spleen when comparing proximal and distal embolization. Additionally, an advantage of proximal embolization is that the procedures can be completed more swiftly, which is crucial for trauma patients whose blood pressure can fluctuate rapidly [119]. Nevertheless, this method is not appropriate for every aneurysm. There are various treatment options available, including basic vascular ligation performed through an open or laparoscopic method, as well as splenectomy if the aneurysm is near the spleen [120].

### 6.2. Cerebral Artery Aneurysms in Pregnancy

#### 6.2.1. Clinical Significance

Intracranial aneurysm is a complicated form of cerebrovascular disease that includes a localized dilatation of the brain’s arteries, which may rupture and result in severe consequences such as subarachnoid hemorrhage. About 3% of the population is affected by them. The specific interaction between these factors is still being studied, even though variables such as genetics and high blood pressure play a role in their development. Due to their location close to the brainstem, basilar artery aneurysms are particularly challenging to treat [121]. The size, location, and form of an aneurysm are used to categorize it. Their size is classified as small (<7 mm), medium (7–12 mm), large (13–25 mm), and giant (>25 mm). In terms of location, they are classified as anterior circulation aneurysms, which are more common, and posterior ones, like basilar artery aneurysms, which have a greater chance of rupturing [122].

#### 6.2.2. Pathophysiology

The weak, bulging wall of a blood vessel can be life-threatening if left untreated in case of an aneurysm. Secondary aneurysms can result from disorders like autoimmune diseases, vasculitis, or infections; whereas, primary cases are often caused by genetic or degenerative factors. High eosinophil levels may indicate that eosinophilic inflammation plays a role in the etiology of some patients [123]. Pregnancy can cause modifications in hormone levels, particularly increases in estrogen and progesterone, as well as changes in blood volume and cardiac output, all of which can cause artery walls to become weaker. These hormones influence collagen and elastin, raising the possibility of aneurysm development or rupture. Rupture of cerebral aneurysms is rare during pregnancy, but it is dangerous and associated with a maternal mortality rate of 5–12% [124].

#### 6.2.3. Risk of Rupture

The location of intracranial aneurysms affects the risk of rupture, but the precise link is still up for debate. The majority of reported cases of ruptured aneurysms occur at the anterior communicating artery, which makes up between 26 and 34% of all cases, indicating that this location may be at a greater risk of rupture. In contrast, studies of unruptured aneurysms frequently discover that the middle cerebral artery (MCA) is the most prevalent site, leading us to wonder if aneurysms in various locations have an intrinsic propensity to rupture [124]. Despite the annual risk of intracranial aneurysm rupture being rather low (between 0.7 and 0.95%), it increases dramatically with the aneurysm’s size. With hazard ratios increasing significantly, large (10–24 mm) and giant (≥25 mm) aneurysms pose a far greater risk of rupture, supporting the necessity for therapy. Although older patients tend to have bigger aneurysms, treatment is difficult because there are more risks involved in the procedure. Improvements in therapy have resulted in better outcomes, even as the aging population increases the incidence of intervention in older patients [125].

#### 6.2.4. Imaging Considerations

Angiography during pregnancy brings up concerns about how radiation may affect the fetus, with the level of risk altering depending on the pregnancy stage—starting from potential loss of the embryo in the beginning to possible developmental issues or brain effects later. Nonetheless, radiation exposure to the fetus during brain imaging is extremely low because of the slight scatter radiation [126]. Fetal radiation exposure from a mother’s head CT scan is very low since the uterus is not in the direct path of the radiation and protective measures are taken. Furthermore, iodinated contrast materials are not linked to teratogenic consequences in late pregnancy, but their usage should be justified. The current recommendations highlight that essential imaging should not be delayed because postponing a diagnosis might increase risks for both the mother and the fetus [127]. Advantages of CT for identifying maternal diseases typically outweigh any potential fetal risks, if there is a clinical need. Nonetheless, alternatives like MRI, which do not use ionizing radiation, are preferred when feasible because CT involves a higher level of radiation exposure. It is crucial to remember that radiation doses less than 100 mGy have not been associated with negative fetal outcomes, and that imaging choices should always prioritize minimizing exposure while maintaining a high level of diagnostic accuracy [128].

#### 6.2.5. Endovascular Treatment

The main goal of aneurysm therapy is to avoid rupture. The two primary approaches are neurosurgical clipping (NC) and endovascular coiling (EC), and the decision is based on different factors including the patient’s age, the characteristics of the aneurysm, whether it has ruptured, and the available resources [129]. NC entails surgically exposing the skull and placing a clip over the aneurysm’s neck to completely stop the blood flow. Although it is more lasting and less likely to recur, it has greater hazards, such as ischemia, vessel damage, and brain injury [130]. Coiling is a less invasive alternative. To fill the aneurysm with coils, a catheter is inserted via the bloodstream (typically via the femoral artery). This encourages clot formation and lowers the chance of rupture. It is particularly helpful in treating aneurysms of the posterior circulation, which are more difficult to treat surgically. Generally speaking, coiling has fewer complications, but it is also more likely to result in incomplete closure and the possibility of recurrence [131]. One study shows that coil embolization had fewer complications than clipping (9.5% vs. 23.1%) in cases of ruptured aneurysms. Surgery decreased complications in unruptured cases by 31.9% when compared to no treatment [132].

Emerging flow-diverting stents show promise for complex cerebral aneurysms, although thrombosis and unpredictable hemodynamics currently limit their use in pregnancy [133].

### 6.3. Comparative Perspective and Role of IR

Visceral and cerebral aneurysms in pregnancy differ significantly in terms of anatomical location, rupture risk, and clinical management, which has direct implications for interventional radiology practice (Table 4). Visceral aneurysms most frequently involve the splenic artery, accounting for approximately 60% of cases, but may also occur in hepatic, mesenteric, or renal arteries [96]. In contrast, cerebral aneurysms arise in intracranial vessels, most commonly within the internal carotid and communicating arteries [134].

Pregnancy exerts a markedly different effect on these entities. Visceral aneurysms are associated with a substantially increased risk of rupture, particularly in the third trimester and in multiparous women [135]. Conversely, although hormonal and hemodynamic changes may contribute to aneurysm formation or growth in the cerebral circulation, the overall risk of rupture appears similar to that observed in the general population [136].

The clinical consequences of rupture also differ considerably. Ruptured visceral aneurysms are associated with extremely high maternal mortality (up to 70–75%) and fetal mortality (up to 90–95%) [135]. In comparison, rupture of a cerebral aneurysm, typically presenting as aneurysmal subarachnoid hemorrhage, carries lower maternal mortality (approximately 5–21%) and fetal mortality around 8–10% [137].

Imaging and treatment strategies reflect these differences. Ultrasound is often the first-line modality for visceral aneurysms, supplemented by CT, MRI, or angiography when necessary [138]. In cerebral aneurysms, CT and MRI are essential for hemorrhage detection, with digital subtraction angiography as the diagnostic gold standard [139]. Endovascular techniques play a central role in both groups, including embolization or stent-grafts for visceral aneurysms and coiling for cerebral aneurysms [117,129].

Given the high rupture risk and mortality, visceral aneurysms are typically managed aggressively during pregnancy, even when small; whereas, cerebral aneurysms require individualized decision-making, with endovascular treatment considered safe when clinically indicated [135].

**Table 4:** Key IR-relevant differences between visceral and cerebral aneurysms in pregnancy.

Feature	Visceral Aneurysm	Cerebral Aneurysm	References
Typical location	Mainly splenic (~60%); also hepatic, mesenteric, renal	Intracranial arteries (ICA, AComA, PComA, basilar)	[96,134]
Relation to pregnancy	Markedly increased rupture risk, especially 3rd trimester	Possible increased formation/growth; rupture risk ~general population	[135,136]
Maternal mortality (rupture)	Very high (up to 70–75%)	Lower (≈5–21%)	[135,137]
Fetal mortality (rupture)	Very high (up to 90–95%)	Lower (≈8–10%)	[135,137]
First-line imaging	US; CT/MR/angiography if needed (with precautions)	CT/MR; DSA for definitive diagnosis	[138,139]
Endovascular treatment	Embolization (coil/plug), stent-graft, vessel sacrifice	Coiling ± stent/balloon	[117,129]
Management in pregnancy	Aggressive; treat even small aneurysms	Individualized; endovascular feasible and safe	[134,135]

ICA, internal carotid artery; AComA, anterior communicating artery; PComA, posterior communicating artery; DSA, digital subtraction angiography; US, ultrasound.

## 7. Palliative and Preoperative Embolization in Gynecological Malignancies

### 7.1. Clinical Significance of Hemorrhage in Gynecological Malignancies

Interventional radiology plays a crucial role in managing patients with advanced stages of gynecological malignancies. The most common gynecological malignancies worldwide include cervical, endometrial, and ovarian cancers, which are responsible for a large number of cancer diagnoses and mortality among women. Among their clinical manifestations, abnormal vaginal bleeding is one of the most frequent symptoms, particularly in cervical and endometrial cancer. Advanced stages of these malignancies may lead to severe hemorrhage requiring urgent intervention [140]. Bleeding associated with gynecological cancers may occur as a consequence of tumor necrosis, invasion of blood vessels or may be linked to surgery and oncological treatment including chemotherapy [141]. The bleeding related to gynecological malignancies is a clinically relevant problem, as it often leads to deterioration of the patient’s general condition, including anemia and episodes of hemodynamic instability, which may require hospitalization and blood transfusions. These complications can cause delays or interruptions in planned cancer treatment, including surgery, chemotherapy, or radiotherapy. This may worsen disease control and make treatment planning more difficult. Therefore, it is important to manage the bleeding as quickly as possible, so the treatment can be continued which optimizes patients outcomes [142,143].

Although hemorrhage is a major complication it can be manageable by minimally invasive interventional radiology methods. These interventions may be helpful in managing symptoms, reduce pain and enhance the possibility of surgical treatment which improve patients’ quality of life. Common image-guided interventional radiology procedures in gynecological oncology include fluid aspiration, drainage catheter placement, and transarterial embolization [141].

### 7.2. Imaging and Patient Selection

Imaging is essential for finding bleeding complications and planning interventional radiology treatment in patients with gynecological cancers. Transabdominal and transvaginal ultrasound are good initial tests for the diagnosis of gynecological conditions including fluid collections or tumor masses; however, more advanced techniques are often required to avoid incorrect diagnosis of clinically important abnormalities. CT and MRI may provide better and more detailed images that are helpful to localize the tumor, its spread, and identify bleeding sources [144]. CT images can show active bleeding or vascular damage but also present the blood supply which is useful for planning embolization [145]. CT angiography is also a useful tool in cases of acute hemorrhage, as it is helpful to identify active bleeding, especially in patients with hemodynamic instability [146]. In some cases of acute pelvic pain with genital bleeding MRI should be chosen instead of CT for further characterization of uterine abnormalities. MRI is also useful to explain abnormal ultrasonography findings before hysteroscopy, as it provides superior detection and of suspicious changes in the uterine cavity. What is more, MRI is considered the best imaging technique for soft tissue characterization and is especially valuable to observe locally advanced tumors and their relationship to surrounding pelvic structures. MRI also avoids the use of ionizing radiation [144,147].

Patients considered for embolization should be examined by a gynecologist and an interventional radiologist. Patient selection for interventional radiology is based on clinical presentation, imaging findings, her interest in future childbearing and overall condition [148]. The candidates typically include patients with advanced stages of genital malignancy or bleeding that occur frequently. These patients are often not suitable for surgical management or have contraindications for the surgery. It is also crucial to determine if the patient already has been treated, and, if so, what type of treatment was performed. The proper preparation before the procedure is essential to improve the success of surgery and reduces the risk of complications [141,145,149]. The embolic material is chosen based on the bleeding cause, its extent, and the number of affected vessels. Proper pain management also contributes to lowering the risk of complications and makes the procedure easier for the patient to tolerate. When the procedure is performed correctly, this type of management offers fast relief of symptoms and supports patient recovery in a minimally invasive and less painful way [148].

**Table 5:** Summary of indications, techniques, and outcomes of palliative and preoperative embolization in gynecological malignancies.

Feature	Palliative Embolization	Preoperative Embolization	References
**Primary goal**	Advanced gynecological malignancies with active bleeding	Hypervascular pelvic tumors planned for surgical resection	[141,145,150]
**Main indication**	Hemostasis, reduced transfusion need, improved quality of life	Reduced intraoperative blood loss and improved surgical field	[151,152]
**Expected benefit**	Enables radiotherapy, chemotherapy, or delayed surgery	Facilitates tumor resection and may reduce operative complexity	[153,154]
**Limitations**	Risk of rebleeding, limited long-term oncologic data	Limited evidence, mostly retrospective studies and case reports	[155,156]

### 7.3. Palliative Embolization

Palliative embolization is a minimally invasive option for tumor-related hemorrhage in advanced gynecological malignancies, especially when surgery is not feasible or rapid stabilization is needed to continue oncologic care [150,157]. Vaginal bleeding in this setting is often driven by local tumor invasion, angiogenesis, treatment effects, and systemic frailty, and it can cause severe anemia, hemodynamic instability, transfusion need, and major deterioration in quality of life [158,159].

The procedure is typically performed through percutaneous arterial access with angiographic identification of bleeding or tumor-feeding vessels, followed by selective or super-selective catheterization and embolization [150,151]. Bilateral treatment is often favored in cervical cancer because collateral pelvic circulation is common, and angiography may reveal multiple bleeding arteries or non-uterine contributors [160,161]. Embolic materials vary by anatomy and operator preference and include gelatin sponge, polyvinyl alcohol particles, microspheres, coils, and drug-eluting beads. Broader cancer data suggest worse outcomes with coils when embolization is proximal rather than super-selective [39,162].

Short-term hemostatic efficacy is consistently high, particularly in cervical cancer. In two retrospective series of locally advanced cervical cancer, bleeding control after first embolization was 95.2% and 95.7%, with no major complications in the larger cohort [151,154]. A larger comparative cohort found pelvic artery embolization outperformed vaginal packing for overall hemostasis (94.0% vs. 57.8%) and reduced recurrence (3.3% vs. 10.4%) [155]. In a multicenter mixed-cancer cohort, transcatheter arterial embolization achieved 89.1% technical success and 84.8% clinical success, although 30-day bleeding-related mortality remained 15.2%, reflecting the severity of underlying malignancy [162]. Smaller uterine body cancer data are less durable: one series reported 100% technical success but only 50% clinical success beyond one week [150].

Embolization also appears to reduce transfusion burden, shorten admission, and allow patients to proceed to radiotherapy or chemotherapy [158,160]. In one cervical cancer cohort, 87.2% of patients required transfusion before embolization, underscoring the acuity of presentation [154]. Palliative hemostatic radiotherapy is another option, with complete bleeding cessation in 80.0% after a median 16 days, but it acts more slowly than embolization and is less suited to immediate hemorrhage control [142].

Complications are usually minor, most often pelvic pain, fever, nausea, and post-embolization syndrome [154,163]. Rare but important adverse events include bladder or rectal ischemia, fistulas, abscess, sepsis, tissue necrosis, and sexual dysfunction [151,164]. Important uncertainties remain: outcome definitions are inconsistent across studies, access is limited to tertiary centers, and one weighted retrospective cohort associated pre-treatment embolization with worse overall survival, although causality is unclear [151,158].

Overall, palliative embolization appears to be an effective, rapid, and generally safe method for controlling severe bleeding in advanced gynecological malignancies, but better comparative studies and clearer long-term oncologic data are still needed [151,159].

### 7.4. Preoperative Embolization

Preoperative embolization aims to devascularize hypervascular pelvic tumors before surgery, improve exposure, and reduce intraoperative hemorrhage, but its benefits vary substantially by indication and tumor type [165,166]. Direct gynecologic evidence for this approach remains limited; as a non-gynecologic extrapolative comparator, the strongest pooled evidence-in head and neck tumors-shows lower blood loss after embolization, although heterogeneity was high and complications were not reduced [165].

In gynecology, the most studied setting is fibroid surgery rather than malignancy. Retrospective data suggest preoperative uterine artery embolization before myomectomy can lower major blood loss and transfusion risk in women with large or multiple fibroids [167]. Another cohort found markedly lower mean blood loss with preoperative UAE, but no improvement in transfusion or hysterectomy rates and signals for worse fertility and pregnancy outcomes [168]. Other studies found no significant reduction in blood loss, underscoring inconsistent effectiveness across series [169]. A 2025 scoping review concluded that evidence for staged UAE before hysterectomy remains too limited for standard-of-care adoption [156].

Evidence in gynecologic malignancy is narrower and mainly case-based. In bleeding cervical cancer, uterine artery embolization can achieve rapid hemostasis and allow subsequent radiotherapy or surgery [152,170]. In a cohort of 81 patients with hemorrhagic cervical cancer, embolization stopped bleeding in 94% and enabled further antitumor treatment in 68% of primary cases [153]. Case reports also describe safer resection of hypervascular vulvar tumors after embolization [171]. By contrast, as a further non-gynecologic extrapolative comparator, propensity-matched studies of hypervascular spinal metastases found no blood-loss benefit and longer operations after preoperative embolization [172].

Overall, preoperative embolization appears most useful in selected hypervascular or hemorrhagic pelvic tumors, especially when surgery would otherwise be high risk, but the literature remains largely retrospective and disease-specific [173,174].

### 7.5. Limitations and Future Perspectives

Current evidence on embolization in gynecological malignancies is constrained primarily by retrospective study designs, which introduce selection bias and limit causal inference across most published series [154,155]. The literature also remains dominated by small cohorts and case-based reports, including studies of 6, 21, and 33 patients, which restrict statistical power and external validity [150,151,158].

In addition, patient populations are heterogeneous with respect to tumor type, stage, prior treatment, bleeding severity, and embolization technique, while outcome definitions and assessment time points are inconsistent [150,155,158]. Although some reports include follow-up, long-term oncologic and hemostatic outcomes remain insufficiently characterized, and the impact of embolization on survival or recurrence is still uncertain [154,155,175]. Future research should prioritize prospective multicenter studies, standardized procedural and outcome-reporting protocols, and robust evaluation of long-term rebleeding, survival, and treatment-integration outcomes [155,159,176].

## 8. Interventional Management of Cesarean Scar Pregnancy

### 8.1. Epidemiology and Clinical Significance

Cesarean scar pregnancy (CSP) is a unique kind of ectopic pregnancy where the fertilized egg attaches itself to the scar from a past cesarean delivery. CSP is distinct from, but shares overlapping imaging features and endovascular management principles with, cervical ectopic pregnancy, another rare form of ectopic implantation [177]. This condition frequently results from inadequate healing of the cesarean incision or irregular growth of the fertilized egg. As the number of cesarean deliveries has grown significantly, the occurrence of CSP has also increased. The rate of CSP varies between 1 in 1800 and 1 in 2216 [178,179]. Gravidity, the count of earlier live births, the tally of past consecutive cesarean sections, and the total number of surgical abortions showed a strong link to the occurrence of CSP in women who have had a cesarean delivery before. This relationship held true across all CSP subtypes. Importantly, the gap between two pregnancies was also significantly linked to CSP occurrence, though this was only noted in type 2 CSP. On the other hand, maternal age did not emerge as an independent risk factor for developing CSP. Beyond having a previous cesarean section, the number of surgical abortions also plays a role in the onset of CSP [180]. Most instances are identified during the initial trimester. If the pregnancy goes past the first trimester, the chances of uterine rupture, bleeding between the mother and fetus, the need for a hysterectomy to control bleeding, and bladder infiltration by an implanted placenta heighten [179,181]. The CSP can pose risks for women due to associated issues like placenta previa or accreta, uterine rupture, and bleeding, which can result in higher rates of illness and death among mothers [182].

### 8.2. Imaging Diagnosis

Transvaginal ultrasound is the primary imaging technique for identifying ectopic pregnancy in cesarean scars, but its effectiveness can vary based on the operator’s skills. This imaging method allows for the assessment of the blood flow to the fetus and the identification of different feeding blood vessels in CSP patients, aiding in the evaluation of bleeding risks, which is crucial for determining the prognosis. Nevertheless, pinpointing the precise location of the pregnancy sac and measuring how deep it has embedded into the muscle layer can sometimes be challenging [183]. In 2000, Vial and colleagues indicated that cesarean scar ectopic pregnancies can be classified into endogenic or exogenic types based on their ultrasound characteristics [184]. Since that time, various classification systems for this condition have been introduced. Nevertheless, these classification systems have not provided numeric ultrasound measurements related to the risk factors for bleeding during the surgical treatment of cesarean scar ectopic pregnancies, nor have they offered particular clinical treatment recommendations according to the classification type [185,186]. On the other hand, MRI offers comprehensive imaging capabilities across various parameters, planes, and directions, along with superior soft tissue clarity. MRI enhances the effectiveness of treatment by detailing how deeply trophoblastic tissue penetrates the myometrium and any possible harm to the serosa or bladder, in addition to pinpointing the precise position of the gestational sac [181]. A study conducted by Xiao and colleagues indicated that MRI outperformed ultrasound in the diagnosis of CSP [183].

Many standards have been suggested for diagnosing CSP, highlighting the necessity for further research into their practical application in a clinical setting. Timor-Tritsch and colleagues suggested that the presence of any of the following indicators could indicate the existence of a CSP:

- Visualization of an empty uterine cavity as well as an empty endocervical canal.
- Detection of the placenta and/or a gestational sac embedded in the hysterotomy scar.
- In early gestations (≤8 weeks), a triangular gestational sac fills the niche of the scar; at ≥8 weeks gestation, this shape may become rounded or even oval.
- A thin (1–3 mm) or absent myometrial layer between the gestational sac and the bladder.
- A closed and empty cervical canal.
- The presence of embryonic/fetal pole and/or yolk sac with or without heart activity.
- The presence of a prominent and at times rich vascular pattern at or in the area of the cesarean scar in the presence of a positive pregnancy test [187].

Early identification is preferable since it simplifies the differentiation between a CSP and a correctly placed pregnancy. Additionally, it provides an opportunity to inform the patient about the possible risks of expectant management and the necessary antenatal monitoring if the pregnancy continues. If the patient decides to terminate the pregnancy, doing so early is linked to fewer complications and lessens the chance of needing a hysterectomy [188].

### 8.3. Role of Uterine Artery Embolization

Termination of pregnancy is usually recommended since cesarean scar pregnancy (CSP) has a high risk of catastrophic consequences such as uterine rupture, abnormal placentation, organ invasion, and life-threatening hemorrhage. It has been shown that uterine artery embolization (UAE) is a safe and efficient treatment strategy when paired with procedures like hysteroscopic resection or curettage. In this situation, UAE lowers uterine blood flow, which decreases the chance of excessive bleeding during later treatment and promotes the gestational sac’s resolution [189]. In the CSP setting specifically, UAE can improve treatment success and reduce the need for additional interventions. Nevertheless, its use may be constrained by high cost, post-procedural pelvic discomfort, and concerns about possible endometrial and ovarian functions. As a result, UAE is utilized more often as an adjuvant therapy, particularly in patients with severe bleeding where surgery is not required [190].

The UAE technique involves selectively catheterizing both uterine arteries and then delivering embolic particles until the desired angiographic endpoint is reached [35]. Under fluoroscopic guidance, uterine artery embolization is carried out using a 5F catheter and femoral artery access. Blood flow occlusion is attained by injecting embolic agents (PVA particles or Gelfoam) into the uterine arteries. Before further ultrasound-guided suction evacuation of the cesarean scar, this decreases trophoblastic vascularization and reduces the risk of bleeding [191]. In clinical practice, both spherical and nonspherical permanent embolic agents, often 500–900 m in diameter, have been thoroughly examined and are widely used [192].

The UAE may be performed as a stand-alone or additional treatment for cesarean scar pregnancy. UAE enhances treatment safety and lowers the risk of bleeding by reducing the gestational sac’s vascular supply. UAE combined with ultrasound-guided dilation and curettage has shown excellent efficacy and uterus preservation; data on long-term reproductive (fertility) outcomes remain comparatively limited, and potential effects on menstrual function and ovarian reserve should be taken into account [193]. Many patients choose UAE because it is a minimally invasive, uterus-preserving alternative to surgery, offering lower morbidity and faster recovery. Especially for women who are more prone to surgical complications like obesity or several intra-abdominal adhesions, it is incredibly valuable [194].

### 8.4. Treatment Outcomes and Uterus Preservation

Suction evacuation, balloon therapy, and surgical removal are among the techniques that may successfully treat Cesarean scar pregnancy (CSP) in more than 90% of cases during the first trimester, based predominantly on retrospective case series rather than randomized comparisons; because the effectiveness of treatment decreases with advancing gestational age, early intervention is advised [195]. CSP can be treated with uterine-preserving procedures that are highly successful; whether reproductive potential is preserved to the same degree as with a physiologically sited pregnancy is less well established, given the shortage of long-term, controlled follow-up data. Medical treatment with methotrexate alone had limited effectiveness; whereas, interventional and surgical methods demonstrated higher success rates [196,197]. Methotrexate, a folate antagonist that inhibits DNA synthesis and cell proliferation, is commonly used for medical management of ectopic pregnancy; however, its effectiveness in CSP is limited, with reported treatment failure rates ranging from 22% to 48% [198]. Because randomized trials in CSP are lacking given the condition’s rarity, guidance on treatment selection rests on international society guidelines and expert consensus statements, themselves derived predominantly from retrospective case series, registry data, and systematic reviews of observational studies rather than randomized comparisons; within this observational evidence base, several treatment options are considered acceptable for CSP, including transvaginal resection, laparoscopy, UAE combined with D&C (with or without hysteroscopy), and hysteroscopic management, with the choice depending on patient condition, symptom severity, available resources, and surgical expertise [199,200]. UAE combined with D&C is a successful strategy that reduces bleeding problems and avoids hysterectomy; nonetheless, the risk of recurrence of CSP and placental abnormalities in future pregnancies necessitates a more thorough assessment of long-term fertility results, and this observational, non-randomized evidence base should be kept in view whenever these approaches are described as “recommended” [201].

In most cases, the uterus can be preserved with conservative CSP care. Following interventional, surgical, and medical procedures, such as UAE, further pregnancies have been documented, although the proportion of women who successfully conceive and deliver after conservative CSP management, relative to those who do not attempt or achieve a subsequent pregnancy, is not well quantified in the available literature. Nevertheless, due to the higher risks of repeated CSP and abnormal placentation in subsequent pregnancies, patients need to be carefully observed [202]. Heterotopic CSP, in which a scar pregnancy coexists with a concurrent intrauterine pregnancy, has also been described and adds further diagnostic and counseling complexity for women with a CSP history [203].

### 8.5. Current Challenges and Future Directions

Due to the rarity of CSP, there is a shortage of randomized research that could provide a clearer understanding of this specific type of pregnancy. Numerous guidelines have been proposed for identifying CSP, emphasizing the need for more investigation into how they can be effectively used in medical practice [187]. Given that most countries do not have clear guidelines for early ultrasounds in women who have had a previous cesarean section, diagnosing through ultrasound becomes more challenging, resulting in many instances likely going undetected, highlighting the necessity for more robust research to thoroughly investigate this condition [204].

## 9. Discussion

### 9.1. Comparative Effectiveness and Safety of the Discussed Procedures

Across the six conditions reviewed, the strength of the underlying evidence, the maturity of comparative data, and the degree of clinical consensus differ substantially, and this heterogeneity should directly temper how confidently each finding is generalized. For symptomatic uterine fibroids, UAE is supported by the most mature evidence base in this review, including multiple randomized trials comparing UAE with surgery; technical success and short-term symptom relief are consistently high, and UAE offers shorter hospital stay, faster recovery, and lower blood loss than myomectomy [205]. This comparative advantage is short-term, however: myomectomy shows better long-term reproductive outcomes (higher clinical pregnancy and live-birth rates, lower miscarriage) [3], and reintervention after UAE is more frequent, particularly with incomplete infarction [2]. Hysterectomy remains the only outcome with zero recurrence risk but at the cost of irreversible loss of the uterus [206]. The principal unresolved controversy for fibroids is therefore not whether UAE works, but which patients should be offered it over myomectomy when future childbearing is a priority—a selection question current evidence cannot fully resolve. In PCS, the evidence for ovarian vein embolization is more limited: no randomized trial has compared embolization with medical therapy or surgery, and the reported 96–100% technical success rate [207] and low reintervention rate [208] derive from cohort series rather than comparative trials. A further, more fundamental controversy-not merely a data-maturity gap-is that the causal relationship between imaging-detected venous insufficiency and chronic pelvic pain is itself contested (Section 4.4); this uncertainty about diagnosis necessarily limits how confidently treatment-effectiveness claims for PCS can be interpreted, independent of any embolization-specific evidence gaps.

In PAS, prophylactic balloon occlusion occupies a fundamentally different evidentiary position: it is an adjunct to, not a substitute for, cesarean hysterectomy, and-unlike UAE for fibroids or embolization for PCS-the core question of whether it reduces hemorrhage compared with surgery alone remains unresolved, with studies reporting decreased transfusion and improved surgical field visualization in some series [209,210] but no consistent hemorrhage reduction across the literature as a whole [90]. Reported complications (arterial thrombosis, ischemic injury, nerve damage) further mean that, unlike the largely favorable risk-benefit balance seen with UAE, the risk-benefit balance for balloon occlusion is not yet established and should be discussed with patients accordingly. In splenic artery aneurysm during pregnancy, the evidence base is the least mature of the four conditions discussed in this subsection: no comparative trial of endovascular versus open repair in pregnancy exists, and the literature consists of case reports and small case series with a plausible publication bias toward successful outcomes. Within that limitation, endovascular embolization is best characterized as a less invasive option that avoids the additional physiologic stress of laparotomy in appropriately selected, hemodynamically stable patients, rather than as a demonstrated safety advantage over open surgery [211,212].

### 9.2. The Role of Interventional Radiology Within a Multidisciplinary Team

A recurring theme across all discussed conditions is the essential role of IR as part of a multidisciplinary team, although the strength of evidence supporting specific multidisciplinary practices, as opposed to the general principle of multidisciplinary care itself, varies. In PAS, multidisciplinary management is the one area in this review with reasonably direct comparative evidence: outcomes are reported to be significantly improved when PAS cases are managed in specialized centers with established multidisciplinary protocols [209,210], so this recommendation is evidence-based rather than purely a matter of consensus. In fibroid management and PCS, by contrast, the case for shared decision-making between interventional radiologists and gynecologists—integrating reproductive goals, symptom severity, imaging findings, and comorbidities—rests on clinical rationale and specialty guidelines rather than on comparative outcome data specifically testing multidisciplinary versus single-specialty pathways [206,207,213]; we characterize this as an expert-consensus recommendation. In SAA and cerebral aneurysm during pregnancy, coordination between obstetricians, interventional or neuro-interventional radiologists, neonatologists or neurosurgeons, and anesthesiologists is likewise supported chiefly by case-series experience and consensus regarding the competing priorities of maternal stabilization, fetal safety, and radiation protection, rather than by comparative trials of coordinated versus uncoordinated care [91,206,214,215]. These examples illustrate that, while IR does not replace surgery but complements it across all six conditions, the evidentiary basis for multidisciplinary care itself ranges from the directly evidence-based (PAS) to reasonable, guideline-supported extrapolation from clinical experience (fibroids, PCS, SAA, cerebral aneurysm).

### 9.3. Limitations of Available Literature and Research Methodology

Despite encouraging results, the available literature has important limitations that affect the strength of the conclusions drawn throughout this review. Many studies assessing UAE, ovarian vein embolization, or balloon occlusion in PAS are retrospective, single-center analyses with relatively small sample sizes, and randomized controlled trials are scarce, particularly in pregnant populations, where ethical and logistical challenges limit study design [38,216]. Heterogeneity represents a major methodological limitation both within and across conditions: differences in patient selection criteria, embolic materials, procedural techniques, operator experience, and outcome definitions make cross-study comparisons difficult even within a single condition, for example fertility outcomes after UAE, which are influenced by patient age, baseline ovarian reserve, fibroid characteristics, and previous reproductive history that are not consistently controlled across studies [50,217]; the same heterogeneity is compounded across this review as a whole by the decision to combine six pathophysiologically distinct conditions—ranging from elective fibroid management to acute obstetric hemorrhage—within a single narrative synthesis, which necessarily limits the extent to which findings from one condition can inform interpretation of another. A further, review-specific limitation is potential selection bias in the 70 guideline, consensus, and background publications added outside the structured screening process (Section 2.1): because these sources were selected by the authors for narrative and contextual relevance rather than through predefined, reproducible criteria, they may disproportionately reflect prevailing expert opinion or the authors’ own prior familiarity with the literature. Publication bias is a further concern, and is plausibly most severe for the acute, emergency interventions covered in this review—balloon occlusion in PAS and embolization for splenic artery aneurysm or acute tumor hemorrhage—where case reports and small series describing successful, dramatic interventions are more likely to be published and cited than unsuccessful or equivocal outcomes, potentially inflating the apparent efficacy of these emergency techniques relative to their true, unpublished failure rate. In PCS research, diagnostic criteria vary widely, and symptom assessment often relies on subjective pain scales without standardized follow-up intervals; studies on prophylactic balloon occlusion in PAS similarly produce conflicting results, partly due to differences in placental invasion severity and institutional expertise [51,92]. Follow-up is short in many reports, particularly regarding long-term fertility and ovarian function, and many recommendations throughout this review are based on observational data and expert consensus rather than high-level evidence, a limitation that is most pronounced for the rare vascular conditions (visceral and cerebral aneurysms in pregnancy), where the available evidence is largely limited to case reports and small case series [136]. Stronger prospective, multicenter randomized trials—or, where a trial is not feasible for a given rare condition, standardized registries—are necessary to clarify comparative effectiveness and long-term safety across all six conditions discussed in this review.

## 10. Conclusions

IR is now an essential component of modern gynecological and obstetric care, offering effective and relatively safe therapeutic alternatives for selected conditions. Uterine artery embolization, ovarian vein embolization, prophylactic balloon occlusion in PAS, and endovascular treatment of splenic artery aneurysms demonstrate that minimally invasive techniques can, in many situations, complement and sometimes replace conventional surgical treatment. In benign conditions, such as fibroids or PCS, embolization provides a lasting improvement in quality of life while preserving the reproductive organs. In high-risk conditions, including PAS and SAA in pregnancy, endovascular interventions can limit life-threatening hemorrhage and improve the mother’s prognosis. In vascular emergencies such as visceral and cerebral aneurysms in pregnancy, endovascular techniques may be life-saving and constitute an essential component of modern multidisciplinary care. Emerging indications such as cesarean scar pregnancy and gynecological malignancies further demonstrate the versatility of interventional radiology. Although further research is needed into long-term reproductive outcomes, the available data suggest that IR represents a valuable, personalized, and organ-preserving therapeutic strategy in selected clinical situations.

## 11. Future Directions

Despite substantial progress, several challenges must be addressed to optimize the role of IR in gynecology and obstetrics. First, there is a clear need for standardization of indications, patient selection criteria, and procedural techniques. Variability in diagnostic definitions, imaging protocols, embolic materials, and operator experience currently limits comparability between studies and may influence clinical outcomes. Developing unified guidelines and standardized treatment algorithms would improve consistency of care, facilitate training, and enhance patient safety [51,205]. Second, continued development of new endovascular technologies and embolic materials is essential. Advances in microcatheter systems, image-guided navigation, radiation-reduction strategies, and bioresorbable or more precisely calibrated embolic agents may further improve procedural accuracy and minimize complications. Innovations aimed at reducing non-target embolization and preserving ovarian reserve are particularly important for women of reproductive age. Research into tailored embolic particles and combined endovascular–pharmacologic approaches may also expand therapeutic possibilities [38,50,52]. Finally, there is a pressing need for large, multicenter, prospective clinical trials with long-term follow-up. Many current recommendations are based on retrospective or single-center studies with limited sample sizes. High-quality randomized controlled trials are necessary to clarify comparative effectiveness, reproductive outcomes, long-term safety, and cost-effectiveness. International collaboration between interventional radiologists, gynecologists, and obstetricians will be crucial to generate robust evidence and to refine evidence-based guidelines. Strengthening the scientific foundation of these procedures will ultimately ensure safer, more effective, and more personalized care for women with complex gynecological and obstetric conditions [51,52,90]. Future research should also focus on optimizing management strategies and evaluating long-term outcomes of endovascular treatment in rare vascular conditions during pregnancy, including visceral and cerebral aneurysms [218].

## Data Availability

No new data were created or analyzed in this study. Data sharing is not applicable to this article, as all information discussed is available in the cited published sources.

## Abbreviations

The following abbreviations are used in this manuscript:

IR	Interventional radiology
UAE	Uterine Artery Embolization
PCS	Pelvic congestion syndrome
PAS	Placenta accreta spectrum
USG	Ultrasonography
CT	Computed Tomography
MRI	Magnetic Resonance Imaging
VAAs	Visceral artery aneurysms
SAA	Splenic artery aneurysm
TVS	Transvaginal scan
TAS	Transabdominal scan
FIGO	International Federation of Gynecology and Obstetrics
PVA	Polyvinyl alcohol
FE	Fibroid expulsion
HRQoL	Health-related quality of life
AMH	Anti-Müllerian hormone
CPP	Chronic pelvic pain
PVI	Pelvic venous insufficiency
PeVD	Pelvic Venous Disorders
SVP	Systems–Varices–Pathophysiology
TVUS	Transvaginal ultrasonography
TAUS	Transabdominal ultrasonography
FSH	Follicle-stimulating hormone
LH	Luteinizing hormone
E2	Estradiol
DIC	Disseminated intravascular coagulation
MCA	Middle cerebral artery
NC	Neurosurgical clipping
EC	Endovascular coiling
